# New records and geographic distribution of the sympatric zones of unisexual and bisexual rock lizards of the genus *Darevskia* in Armenia and adjacent territories

**DOI:** 10.3897/BDJ.8.e56030

**Published:** 2020-09-16

**Authors:** Varos G Petrosyan, Fedor A Osipov, Vladimir V Bobrov, Natalia N Dergunova, Ivan I Kropachev, Felix D Danielyan, Marine S Arakelyan

**Affiliations:** 1 A.N. Severtsov Institute of Ecology and Evolution of the RussianAcademy of Sciences, Moscow, Russia A.N. Severtsov Institute of Ecology and Evolution of the RussianAcademy of Sciences Moscow Russia; 2 Tula Exotarium, Tula, Russia Tula Exotarium Tula Russia; 3 Department of Biology, Yerevan State University, Yerevan, Armenia Department of Biology, Yerevan State University Yerevan Armenia

**Keywords:** Reptilia, reticulate evolution, parthenogenesis, Caucasian rock lizards, bisexual species, parthenogenetic species.

## Abstract

**Background:**

Caucasian rock lizards of the genus *Darevskia* are unique taxa, including both bisexual and parthenogenetic species. The parthenogenetic species have originated as a result of natural hybridisation between females and males of different bisexual species. The species involved in interspecific hybridisation are called parental. However, sympatric zones (SZ) of unisexual and bisexual rock lizards of the Caucasus are still poorly studied, although they are very important for understanding the role of hybrid individuals of different origin in reticulate evolution. This paper presents the location of the SZs of parthenogenetic and their parental bisexual rock lizards of the genus *Darevskia* in Armenia and adjacent territories of Georgia and Nagorno-Karabakh. We summarised the locations of the SZs identified from 1957 to the present, based on our field survey data gathered in 2018-2019 and records from publications and museum collections. This dataset includes 39 SZs of three types: SZ of parental bisexual species, SZ of parental species with unisexual species and SZ of the parthenogenetic species. For each zone, species composition, geographical and altitudinal distribution are presented. New records expand our knowledge of the geographical and altitudinal distribution of SZs in these species and provide additional data for understanding the mechanisms of reticulate evolution and hybridogeneous speciation in the past, present and future.

**New information:**

The new records, including geographical and altitudinal distributions of three types of SZs, are presented, which expand the previously-known list to 39 locations of contact zones for parthenogenetic and its bisexual parental species of rock lizards of the genus *Darevskia* in Armenia and the adjacent territories of Georgia and Nagorno-Karabakh.

## Introduction

Caucasian rock lizards of the genus *Darevskia* (Lacertidae) are the first group of terrestrial vertebrates, in which true parthenogenesis was discovered (Darevsky 1958). At present, it has been found that parthenogenesis in reptiles is known in less than 0.46% of species (Fujita and Moritz 2009). In general, cytological, genetic, morphological and ecological studies have shown that parthenogenetic lizards of the genus *Darevskia*, namely *D. dahli* (Darevsky, 1957), *D. rostombekowi* (Darevsky, 1957), *D. uzzelli* (Darevsky et Danielyan 1977), *D. armeniaca* (Méhely, 1909), *D. unisexualis* (Darevsky, 1966), *D. sapphirina* (Schmidtler et al., 1994) and *D. bendimahiensis* (Schmidtler et al., 1994) have originated as a result of natural hybridisation between bisexual parental species (Darevsky 1958, Darevsky 1967, Uzzell and Darevsky 1975, Macculloch et al. 1997, Fu et al. 1999, Fu et al. 2000a, Fu et al. 2000b, Murphy et al. 2000, Kupriyanova 1999, Martirosian et al. 2002, Martirosian et al. 2003, Malysheva et al. 2007a, Malysheva et al. 2007b).

It has been established that only four parental bisexual species were involved in the origin of clonal forms: the females of *D. raddei* (Boettger, 1892) and *D. mixta* (Méhely, 1909) as "maternal" species and the males of *D. valentini* (Boettger, 1892) and *D. portschinskii* (Kessler, 1878) as "paternal" species (Fu et al. 1997, Murphy et al. 2000). The bisexual species *D. raddei* most often participated in hybridisation. This species is “maternal” for at least five parthenogenetic forms — *D. unisexualis*, *D. uzzelli*, *D. bendimahiensis*, *D. sapphirina* and *D. rostombekowi* (Fu et al. 1997). *Darevskia raddei* is considered as a species complex (*Darevskia raddei* sensu lato) containing four forms of “*raddei*”, “*nairensis*”, “*vanensis*” and “*chaldoranensis*” (Grechko et al. 2007, Rastegar-Pouyani et al. 2012, Omelchenko et al. 2016). "Paternal" species belong to the clade "rudis" (*D. valentini* and *D. portschinskii*) and "maternal" species - "caucasica" (*D. mixta and D. r. raddei*). The population density of parthenogenetic species is often greater than in bisexual species (Darevsky 1967, Galoyan 2010, Tarkhnishvili et al. 2010). However, there are sufficient numbers of SZs of parthenogenetic species, as well as SZs of unisexual forms with their parental species. In some of these SZs, there is crossbreeding between bisexual and parthenogenetic species (Danielyan et al. 2008). In addition, karyological analysis showed that some intermediate large specimens of hybrids are triploids (3n) or tetraploids (4n) (Danielyan et al. 2008). The theory of sequential polyploidisation indicates that hybridisation (Borkin and Darevsky 1980), parthenogenesis and polyploidy are means of rapid speciation (Darevsky et al. 1985, Kearney et al. 2009). Therefore, parthenogenesis can be considered as some adaptive strategy in reptiles, which can affect the structure of niches and, possibly, affect parental species. One of the important challenges in studying the ecology and evolution of parthenogenetic forms is to identify SZs of co-existence of bisexual and unisexual lizards. In particular, it was shown that many of the 112 females of the analysed species *D. armeniaca* and the "paternal" species *D. valentini* from the SZ Kuchak (Armenia) were characterised by copulation marks (Carretero et al. 2018). Copulation marks, of course, do not necessarily lead to copulation; however, between-species copulations were observed regularly (Galoyan 2013). Females of all species showed copulation marks with a frequency ranging from 80% in *D. valentini* to 64% in *D. armeniaca*. From these, seven of eleven (64%) backcross females also showed copulation marks. *Darevskia valentini* males showed no evidence of marks, while marks from all analysed females were exclusively found in the flank, i.e. the inguinal region, which is characteristic of the clade "rudis" (Darevsky 1967, Murphy et al. 2000). The intensity of copulation marks increased with increasing body size of the most abundant parthenogenetic species *D. armeniaca* in the study of SZ Kuchak. These results show that copulation between parthenogenetic and bisexual species in mixed *Darevskia* communities is widespread and driven by sexual selection, which confirms previous assumptions about reproductive interaction in SZs.

Unlike numerous triploid hybrids (*D. armeniaca* × *D. valentini*, *D. unisexualis* × *D. valentini*, *D. dahli* × *D. portschinskii*) that appear in sympatric populations between parthenogenetic and bisexual species, a rare male was caught in the “pure” *D. armeniaca* populations (vicinity of Stepanavan, Armenia) (Darevskii and Kupriyanova 1982). A study of the extensive material, collected at different times, made it possible to reveal the existence of "parthenogenetic males", whose occurrence in nature does not exceed 0.1% (Darevsky et al. 1978, Darevskii and Kupriyanova 1982). It was revealed that these males differ from parthenogenetic females with a relatively larger head and a brighter green colour on the upper side of the body. Their male affiliation was also proved by the presence of characteristic genitals and apparently fully-developed testes. It is worth noting that Dobrowolska (Dobrowolska 1964) and Darevsky (Darevsky 1966) also studied two males of *D. dahli*, which outwardly did not differ completely from females of the same species. Although the appearance of males in parthenogenetic populations is a rare event, nevertheless, they can be important in reticulate evolution and require further studying (Danielyan 1970). Several rare parthenogenetic males were also previously found in two species of North American parthenogenetic lizards, *C. tessellatus* and *C. velox* of the genus *Cnemidophorus* (Maslin 1962).

Thus, it is not excluded that the spontaneous appearance of the hybrid males in the SZs of parthenogenetic lineages and their further hybridisation with females of parental species can give rise to the emergence of new parthenogenetic forms, i.e. the possibility of the emergence of contagious parthenogenesis (Maccari et al. 2014). Contagious parthenogenesis is a process in which rare functional males, produced by the parthenogenetic lineages, mate with co-existing bisexual females, leading to fertile parthenogenetic offspring. This is one of the most striking mechanisms responsible for the generation of new parthenogenetic lineages.

In order to understand the frequency of occurrence of new hybrid forms and the type of interspecific mating of rock lizards in SZs, their inventory and documentation are required. This study is aimed at studying the geographical distribution of SZs' Caucasian rock lizards of the genus *Darevskia*, as potential sites of new parthenogenetic (2n), triploid (3n) and tetraploid (4n) forms, which play an important role in understanding the theory of reticulate evolution.

## Materials and methods

The materials for creating the set of SZs' records and their geographical distribution were data of parthenogenetic and bisexual species collected by the authors during field studies from 1967-2017, as well as data from additional field surveys carried out in June and July in 2018 and 2019. New SZs were found and the coordinates of those only previously known by the name of the settlements zones were specified for the first time in 2018-2019. During the field survey, we registered all zones on routes over a total length of 4800 km. At each site, the group conducted studies lasting up to 1 hour to confirm or reject the presence of the studied species of lizards. Field surveys were carried out in sunny morning windless hours to reduce the influence of weather on the activity of lizards. For each site, geographical coordinates (longitude, latitude) and altitude (above sea level) were determined using the Garmin Montana 680t GPS receiver (Garmin Corp., Olathe, KS, USA) in 2018- 2019 and the coordinates of species occurrence sites recorded in other years were determined using Magellan Spor Trak, Garmin Decota 10, Garmin GPS Map 64. Geographic coordinates were determined with an accuracy of ± 3.5 m.

**Figure 1.**
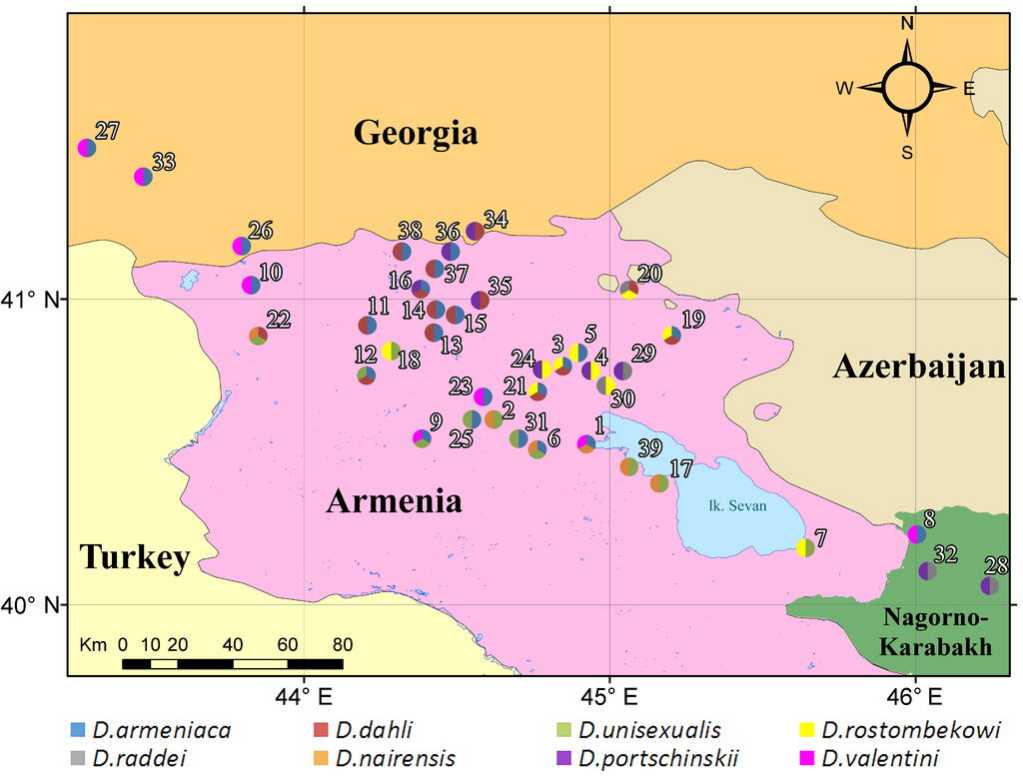
Geographic distribution of SZs in the Armenia and adjacent territories of Georgia and Nagorno-Karabakh, based on our new field survey data, museum and literature records. SZs are indicated by numbers from 1 to 39.

**Figure 2.**
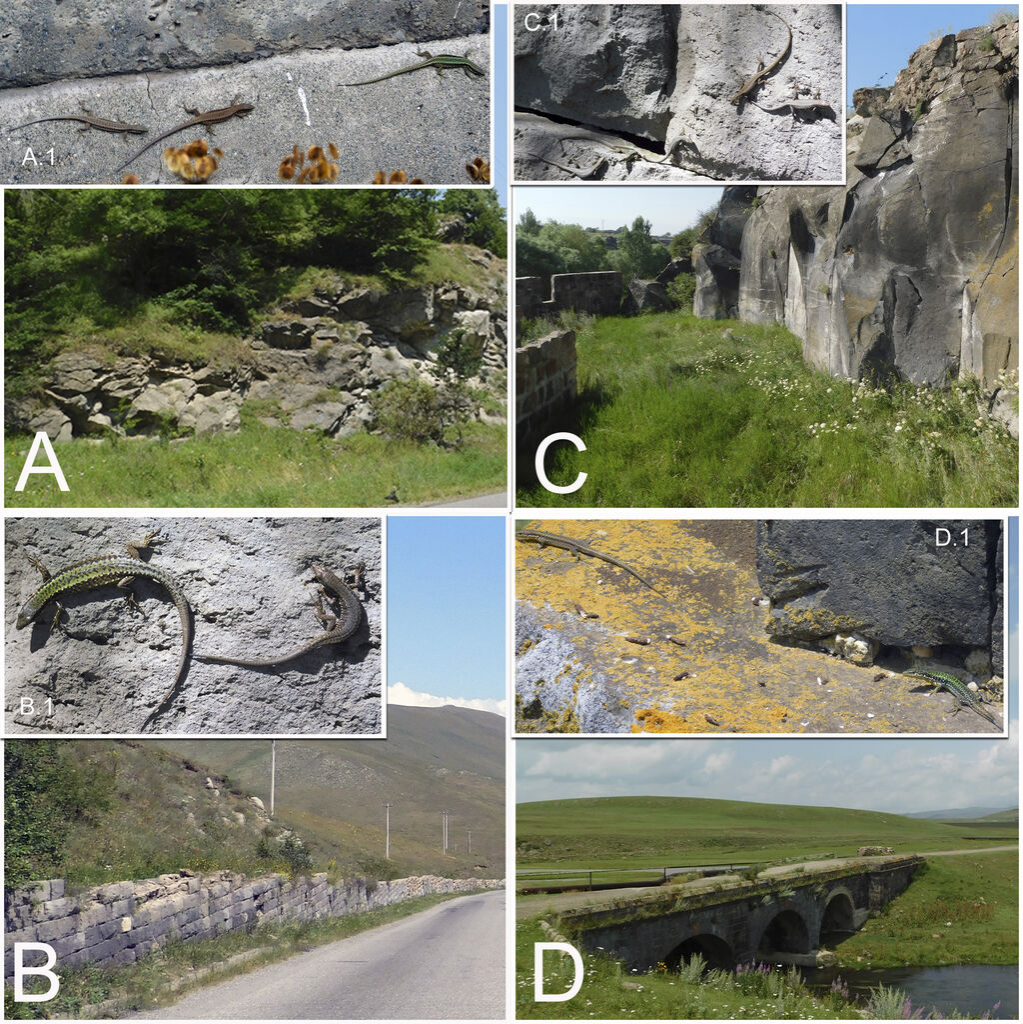
Four typical sympatric zones of lizards in north-eastern and north-western parts of Armenia, where **A.** Dilijan (SZ № 3 of species: *D. armeniaca*, *D. dahli*, *D. rostombekowi*); **A.1.** *D. armeniaca*, *D. dahli* and *D. rostombekowi* on the rock; **B.** Tsilkar (SZ №12 of species: *D. unisexualis*, *D. armeniaca*, *D. dahli)*; **B.1.** *D. dahli* and *D. armeniaca* on the stone wall; **С.** Keti vicinity (SZ № 22 of species: *D. dahli*, *D. unisexualis*, *D. nairensis*); **C.1.** Group of *D. dahli* and *D. unisexualis* on the stone wall; **D.** Sepasar (SZ №10 of species: *D. armeniaca*, *D. valentini*); **D.1.** *D. armeniaca* and *D. valentini* on the stone bridge. Photos by F. Osipov.

During the field studies, specimens were captured by noose. Captured lizards were photographed (an anterolateral surface and the temporal areas of the head, as well as the anal area) with a Nikon Coolpix B500 digital camera to enable further checking of species identification in the laboratory. The sexing of the captured individuals was done by visual inspection. The gender of the rock lizards were easily distinguished by the enlarged femoral pores in the ventral region of the hind legs in males. The males are usually characterised by a larger head and brighter colours, as well as deep blue markings along the side of their belly. In doubtful cases, the lizard was examined to determine the presence of a hemipenis without harming it. Specimens were released at the place of capture immediately after registration. The field survey was carried out under a Scientific Purposes Permit from the Ministry of Nature Protection of the Republic of Armenia Code 5/22.1/51043 for activities pertaining to the capture, handling and/or collection of wild animals for scientific purposes, including Armenian–Russian collaborative projects. During the field survey in 2018-2019, 121 habitats were analysed, 39 SZs were identified and 159 individuals were captured, which represented four parthenogenetic (*D. armeniaca*, *D. dahli*, *D. rostombekowi*, *D. unisexualis*) and four parental species (*D. portschinskii*, *D. r. raddei*, *D. r. nairensis*, *D. valentini*) (Table 1, Fig. 1). Some typical SZs of lizards in north-eastern and north-western parts of Armenia are presented in Fig. 2.

## Taxon treatments

### Darevskia armeniaca

(Mehely, 1909)

30AE7E75-B2CE-5EBC-99BB-E8D02A43CE16

#### Materials

- **Type status:** Other material. **Occurrence:** catalogNumber: REPAMPHRU2018386; recordedBy: Osipov F.A.; individualCount: 1; sex: fmale; lifeStage: adult; occurrenceID: urn:IEERASBIOINF:REPAMPHRU2018386; **Taxon:** scientificName: Darevskia armeniaca; kingdom: Animalia ; phylum: Chordata ; class: Reptilia ; order: Squamata ; family: Lacertidae; genus: Darevskia ; scientificNameAuthorship: Mehely, 1909; **Location:** country: Armenia; stateProvince: Gegharkunik Province; locality: Lchashen, Sevan lake; decimalLatitude: 40.510698; decimalLongitude: 44.935422; geodeticDatum: WGS1984; georeferenceProtocol: GPS; **Identification:** identifiedBy: Arakelyan M.S.; **Event:** samplingProtocol: Captured by noose; eventDate: 2018-7-8; **Record Level:** language: en; rights: https://creativecommons.org/publicdomain/zero/1.0/; rightsHolder: Petrosyan V.G.; accessRights: http://vertnet.org/resources/norms.html; institutionCode: IEERASBIOINF; collectionCode: REPAMPHRU; basisOfRecord: HumanObservation
- **Type status:** Other material. **Occurrence:** catalogNumber: REPAMPHRU2018393; recordedBy: Osipov F.A.; individualCount: 1; sex: fmale; lifeStage: adult; occurrenceID: urn:IEERASBIOINF:REPAMPHRU2018393; **Taxon:** scientificName: Darevskia armeniaca; kingdom: Animalia ; phylum: Chordata ; class: Reptilia ; order: Squamata ; family: Lacertidae ; genus: Darevskia ; scientificNameAuthorship: Mehely, 1909; **Location:** country: Armenia; stateProvince: Tavush Province; locality: Dilijan; decimalLatitude: 40.733998; decimalLongitude: 44.81778; geodeticDatum: WGS1984; georeferenceProtocol: GPS; **Identification:** identifiedBy: Arakelyan M.S.; **Event:** samplingProtocol: Captured by noose; eventDate: 2018-7-9; **Record Level:** language: en; rights: https://creativecommons.org/publicdomain/zero/1.0/; rightsHolder: Petrosyan V.G.; accessRights: http://vertnet.org/resources/norms.html; institutionCode: IEERASBIOINF; collectionCode: REPAMPHRU; basisOfRecord: HumanObservation
- **Type status:** Other material. **Occurrence:** catalogNumber: REPAMPHRU2018400; recordedBy: Osipov F.A.; individualCount: 1; sex: fmale; lifeStage: adult; occurrenceID: urn:IEERASBIOINF:REPAMPHRU2018400; **Taxon:** scientificName: Darevskia armeniaca; kingdom: Animalia ; phylum: Chordata ; class: Reptilia ; order: Squamata ; family: Lacertidae ; genus: Darevskia ; scientificNameAuthorship: Mehely, 1909; **Location:** country: Armenia; stateProvince: Tavush Province; locality: Haghartsin Monastery; decimalLatitude: 40.801931; decimalLongitude: 44.890573; geodeticDatum: WGS1984; georeferenceProtocol: GPS; **Identification:** identifiedBy: Arakelyan M.S.; **Event:** samplingProtocol: Captured by noose; eventDate: 2018-7-9; **Record Level:** language: en; rights: https://creativecommons.org/publicdomain/zero/1.0/; rightsHolder: Petrosyan V.G.; accessRights: http://vertnet.org/resources/norms.html; institutionCode: IEERASBIOINF; collectionCode: REPAMPHRU; basisOfRecord: HumanObservation
- **Type status:** Other material. **Occurrence:** catalogNumber: REPAMPHRU2018401; recordedBy: Osipov F.A.; individualCount: 1; sex: fmale; lifeStage: adult; occurrenceID: urn:IEERASBIOINF:REPAMPHRU2018401; **Taxon:** scientificName: Darevskia armeniaca; kingdom: Animalia ; phylum: Chordata ; class: Reptilia ; order: Squamata ; family: Lacertidae ; genus: Darevskia ; scientificNameAuthorship: Mehely, 1909; **Location:** country: Armenia; stateProvince: Kotayk Province; locality: Hrazdan city; decimalLatitude: 40.506393; decimalLongitude: 44.748776; geodeticDatum: WGS1984; georeferenceProtocol: GPS; **Identification:** identifiedBy: Arakelyan M.S.; **Event:** samplingProtocol: Captured by noose; eventDate: 2018-7-9; **Record Level:** language: en; rights: https://creativecommons.org/publicdomain/zero/1.0/; rightsHolder: Petrosyan V.G.; accessRights: http://vertnet.org/resources/norms.html; institutionCode: IEERASBIOINF; collectionCode: REPAMPHRU; basisOfRecord: HumanObservation
- **Type status:** Other material. **Occurrence:** catalogNumber: REPAMPHRU2018407; recordedBy: Osipov F.A.; individualCount: 3; sex: fmale; lifeStage: adult; occurrenceID: urn:IEERASBIOINF:REPAMPHRU2018407; **Taxon:** scientificName: Darevskia armeniaca; kingdom: Animalia ; phylum: Chordata ; class: Reptilia ; order: Squamata ; family: Lacertidae ; genus: Darevskia ; scientificNameAuthorship: Mehely, 1909; **Location:** country: Armenia; stateProvince: Gegharkunik Province; locality: Karabakh–Sotk road; decimalLatitude: 40.223085; decimalLongitude: 46.00103; geodeticDatum: WGS1984; georeferenceProtocol: GPS; **Identification:** identifiedBy: Arakelyan M.S.; **Event:** samplingProtocol: Captured by noose; eventDate: 2018-7-10; **Record Level:** language: en; rights: https://creativecommons.org/publicdomain/zero/1.0/; rightsHolder: Petrosyan V.G.; accessRights: http://vertnet.org/resources/norms.html; institutionCode: IEERASBIOINF; collectionCode: REPAMPHRU; basisOfRecord: HumanObservation
- **Type status:** Other material. **Occurrence:** catalogNumber: REPAMPHRU2018408; recordedBy: Osipov F.A.; individualCount: 1; sex: fmale; lifeStage: adult; occurrenceID: urn:IEERASBIOINF:REPAMPHRU2018408; **Taxon:** scientificName: Darevskia armeniaca; kingdom: Animalia ; phylum: Chordata ; class: Reptilia ; order: Squamata ; family: Lacertidae ; genus: Darevskia ; scientificNameAuthorship: Mehely, 1909; **Location:** country: Armenia; stateProvince: Aragatsotn Province; locality: Kuchak; decimalLatitude: 40.528691; decimalLongitude: 44.388427; geodeticDatum: WGS1984; georeferenceProtocol: GPS; **Identification:** identifiedBy: Arakelyan M.S.; **Event:** samplingProtocol: Captured by noose; eventDate: 2018-7-11; **Record Level:** language: en; rights: https://creativecommons.org/publicdomain/zero/1.0/; rightsHolder: Petrosyan V.G.; accessRights: http://vertnet.org/resources/norms.html; institutionCode: IEERASBIOINF; collectionCode: REPAMPHRU; basisOfRecord: HumanObservation
- **Type status:** Other material. **Occurrence:** catalogNumber: REPAMPHRU2018411; recordedBy: Osipov F.A.; individualCount: 1; sex: fmale; lifeStage: adult; occurrenceID: urn:IEERASBIOINF:REPAMPHRU2018411; **Taxon:** scientificName: Darevskia armeniaca; kingdom: Animalia ; phylum: Chordata ; class: Reptilia ; order: Squamata ; family: Lacertidae ; genus: Darevskia ; scientificNameAuthorship: Mehely, 1909; **Location:** country: Armenia; stateProvince: Shirak Province; locality: Mets Sepasar; decimalLatitude: 41.030369; decimalLongitude: 43.820932; geodeticDatum: WGS1984; georeferenceProtocol: GPS; **Identification:** identifiedBy: Arakelyan M.S.; **Event:** samplingProtocol: Captured by noose; eventDate: 2018-7-15; **Record Level:** language: en; rights: https://creativecommons.org/publicdomain/zero/1.0/; rightsHolder: Petrosyan V.G.; accessRights: http://vertnet.org/resources/norms.html; institutionCode: IEERASBIOINF; collectionCode: REPAMPHRU; basisOfRecord: HumanObservation
- **Type status:** Other material. **Occurrence:** catalogNumber: REPAMPHRU2018413; recordedBy: Osipov F.A.; individualCount: 2; sex: fmale; lifeStage: adult; occurrenceID: urn:IEERASBIOINF:REPAMPHRU2018413; **Taxon:** scientificName: Darevskia armeniaca; kingdom: Animalia ; phylum: Chordata ; class: Reptilia ; order: Squamata ; family: Lacertidae ; genus: Darevskia ; scientificNameAuthorship: Mehely, 1909; **Location:** country: Armenia; stateProvince: Lori Province; locality: Gogaran; decimalLatitude: 40.895442; decimalLongitude: 44.202444; geodeticDatum: WGS1984; georeferenceProtocol: GPS; **Identification:** identifiedBy: Arakelyan M.S.; **Event:** samplingProtocol: Captured by noose; eventDate: 2018-7-16; **Record Level:** language: en; rights: https://creativecommons.org/publicdomain/zero/1.0/; rightsHolder: Petrosyan V.G.; accessRights: http://vertnet.org/resources/norms.html; institutionCode: IEERASBIOINF; collectionCode: REPAMPHRU; basisOfRecord: HumanObservation
- **Type status:** Other material. **Occurrence:** catalogNumber: REPAMPHRU2019417; recordedBy: Osipov F.A.; individualCount: 6; sex: fmale; lifeStage: adult; occurrenceID: urn:IEERASBIOINF:REPAMPHRU2019417; **Taxon:** scientificName: Darevskia armeniaca; kingdom: Animalia ; phylum: Chordata ; class: Reptilia ; order: Squamata ; family: Lacertidae ; genus: Darevskia ; scientificNameAuthorship: Mehely, 1909; **Location:** country: Armenia; stateProvince: Aragatsotn Province; locality: Tsilkar; decimalLatitude: 40.736893; decimalLongitude: 44.197427; geodeticDatum: WGS1984; georeferenceProtocol: GPS; **Identification:** identifiedBy: Arakelyan M.S.; **Event:** samplingProtocol: Captured by noose; eventDate: 2019-7-16; **Record Level:** language: en; rights: https://creativecommons.org/publicdomain/zero/1.0/; rightsHolder: Petrosyan V.G.; accessRights: http://vertnet.org/resources/norms.html; institutionCode: IEERASBIOINF; collectionCode: REPAMPHRU; basisOfRecord: HumanObservation
- **Type status:** Other material. **Occurrence:** catalogNumber: REPAMPHRU2019419; recordedBy: Osipov F.A.; individualCount: 1; sex: fmale; lifeStage: adult; occurrenceID: urn:IEERASBIOINF:REPAMPHRU2019419; **Taxon:** scientificName: Darevskia armeniaca; kingdom: Animalia ; phylum: Chordata ; class: Reptilia ; order: Squamata ; family: Lacertidae ; genus: Darevskia ; scientificNameAuthorship: Mehely, 1909; **Location:** country: Armenia; stateProvince: Lori Province; locality: Pushkin pass; decimalLatitude: 40.917347; decimalLongitude: 44.436738; geodeticDatum: WGS1984; georeferenceProtocol: GPS; **Identification:** identifiedBy: Arakelyan M.S.; **Event:** samplingProtocol: Captured by noose; eventDate: 2019-7-16; **Record Level:** language: en; rights: https://creativecommons.org/publicdomain/zero/1.0/; rightsHolder: Petrosyan V.G.; accessRights: http://vertnet.org/resources/norms.html; institutionCode: IEERASBIOINF; collectionCode: REPAMPHRU; basisOfRecord: HumanObservation
- **Type status:** Other material. **Occurrence:** catalogNumber: REPAMPHRU2019421; recordedBy: Osipov F.A.; individualCount: 1; sex: fmale; lifeStage: adult; occurrenceID: urn:IEERASBIOINF:REPAMPHRU2019421; **Taxon:** scientificName: Darevskia armeniaca; kingdom: Animalia ; phylum: Chordata ; class: Reptilia ; order: Squamata ; family: Lacertidae ; genus: Darevskia ; scientificNameAuthorship: Mehely, 1909; **Location:** country: Armenia; stateProvince: Lori Province; locality: Pushkin memorial end; decimalLatitude: 40.93278; decimalLongitude: 44.44017; geodeticDatum: WGS1984; georeferenceProtocol: GPS; **Identification:** identifiedBy: Arakelyan M.S.; **Event:** samplingProtocol: Captured by noose; eventDate: 2019-7-16; **Record Level:** language: en; rights: https://creativecommons.org/publicdomain/zero/1.0/; rightsHolder: Petrosyan V.G.; accessRights: http://vertnet.org/resources/norms.html; institutionCode: IEERASBIOINF; collectionCode: REPAMPHRU; basisOfRecord: HumanObservation
- **Type status:** Other material. **Occurrence:** catalogNumber: REPAMPHRU2019423; recordedBy: Osipov F.A.; individualCount: 1; sex: fmale; lifeStage: adult; occurrenceID: urn:IEERASBIOINF:REPAMPHRU2019423; **Taxon:** scientificName: Darevskia armeniaca; kingdom: Animalia ; phylum: Chordata ; class: Reptilia ; order: Squamata ; family: Lacertidae ; genus: Darevskia ; scientificNameAuthorship: Mehely, 1909; **Location:** country: Armenia; stateProvince: Lori Province; locality: Dendropark; decimalLatitude: 40.93852; decimalLongitude: 44.479332; geodeticDatum: WGS1984; georeferenceProtocol: GPS; **Identification:** identifiedBy: Arakelyan M.S.; **Event:** samplingProtocol: Captured by noose; eventDate: 2019-7-16; **Record Level:** language: en; rights: https://creativecommons.org/publicdomain/zero/1.0/; rightsHolder: Petrosyan V.G.; accessRights: http://vertnet.org/resources/norms.html; institutionCode: IEERASBIOINF; collectionCode: REPAMPHRU; basisOfRecord: HumanObservation
- **Type status:** Other material. **Occurrence:** catalogNumber: REPAMPHRU2019425; recordedBy: Osipov F.A.; individualCount: 5; sex: fmale; lifeStage: adult; occurrenceID: urn:IEERASBIOINF:REPAMPHRU2019425; **Taxon:** scientificName: Darevskia armeniaca; kingdom: Animalia ; phylum: Chordata ; class: Reptilia ; order: Squamata ; family: Lacertidae ; genus: Darevskia ; scientificNameAuthorship: Mehely, 1909; **Location:** country: Armenia; stateProvince: Lori Province; locality: Dzoraget; decimalLatitude: 41.014219; decimalLongitude: 44.379631; geodeticDatum: WGS1984; georeferenceProtocol: GPS; **Identification:** identifiedBy: Arakelyan M.S.; **Event:** samplingProtocol: Captured by noose; eventDate: 2019-7-16; **Record Level:** language: en; rights: https://creativecommons.org/publicdomain/zero/1.0/; rightsHolder: Petrosyan V.G.; accessRights: http://vertnet.org/resources/norms.html; institutionCode: IEERASBIOINF; collectionCode: REPAMPHRU; basisOfRecord: HumanObservation
- **Type status:** Other material. **Occurrence:** catalogNumber: REPAMPHRU2019432; recordedBy: Osipov F.A.; individualCount: 1; sex: fmale; lifeStage: adult; occurrenceID: urn:IEERASBIOINF:REPAMPHRU2019432; **Taxon:** scientificName: Darevskia armeniaca; kingdom: Animalia ; phylum: Chordata ; class: Reptilia ; order: Squamata ; family: Lacertidae ; genus: Darevskia ; scientificNameAuthorship: Mehely, 1909; **Location:** country: Armenia; stateProvince: Tavush Province; locality: Serpentine from Ijevan; decimalLatitude: 40.868307; decimalLongitude: 45.187475; geodeticDatum: WGS1984; georeferenceProtocol: GPS; **Identification:** identifiedBy: Arakelyan M.S.; **Event:** samplingProtocol: Captured by noose; eventDate: 2019-7-22; **Record Level:** language: en; rights: https://creativecommons.org/publicdomain/zero/1.0/; rightsHolder: Petrosyan V.G.; accessRights: http://vertnet.org/resources/norms.html; institutionCode: IEERASBIOINF; collectionCode: REPAMPHRU; basisOfRecord: HumanObservation
- **Type status:** Other material. **Occurrence:** catalogNumber: REPAMPHRU2019438; recordedBy: Osipov F.A.; individualCount: 1; sex: fmale; lifeStage: adult; occurrenceID: urn:IEERASBIOINF:REPAMPHRU2019438; **Taxon:** scientificName: Darevskia armeniaca; kingdom: Animalia ; phylum: Chordata ; class: Reptilia ; order: Squamata ; family: Lacertidae ; genus: Darevskia ; scientificNameAuthorship: Mehely, 1909; **Location:** country: Armenia; stateProvince: Lori Province; locality: Fioletova; decimalLatitude: 40.715555; decimalLongitude: 44.77062; geodeticDatum: WGS1984; georeferenceProtocol: GPS; **Identification:** identifiedBy: Arakelyan M.S.; **Event:** samplingProtocol: Captured by noose; eventDate: 2019-7-24; **Record Level:** language: en; rights: https://creativecommons.org/publicdomain/zero/1.0/; rightsHolder: Petrosyan V.G.; accessRights: http://vertnet.org/resources/norms.html; institutionCode: IEERASBIOINF; collectionCode: REPAMPHRU; basisOfRecord: HumanObservation
- **Type status:** Other material. **Occurrence:** catalogNumber: REPAMPHRU2019444; recordedBy: Osipov F.A.; individualCount: 1; sex: fmale; lifeStage: adult; occurrenceID: urn:IEERASBIOINF:REPAMPHRU2019444; **Taxon:** scientificName: Darevskia armeniaca; kingdom: Animalia ; phylum: Chordata ; class: Reptilia ; order: Squamata ; family: Lacertidae ; genus: Darevskia ; scientificNameAuthorship: Mehely, 1909; **Location:** country: Armenia; stateProvince: Kotayk Province; locality: Tezh; decimalLatitude: 40.6545833333; decimalLongitude: 44.5810166667; geodeticDatum: WGS1984; georeferenceProtocol: GPS; **Identification:** identifiedBy: Arakelyan M.S.; **Event:** samplingProtocol: Captured by noose; eventDate: 2018-7-11; **Record Level:** language: en; rights: https://creativecommons.org/publicdomain/zero/1.0/; rightsHolder: Petrosyan V.G.; accessRights: http://vertnet.org/resources/norms.html; institutionCode: IEERASBIOINF; collectionCode: REPAMPHRU; basisOfRecord: HumanObservation
- **Type status:** Other material. **Occurrence:** catalogNumber: REPAMPHRU2019448; recordedBy: Osipov F.A.; individualCount: 1; sex: fmale; lifeStage: adult; occurrenceID: urn:IEERASBIOINF:REPAMPHRU2019448; **Taxon:** scientificName: Darevskia armeniaca; kingdom: Animalia ; phylum: Chordata ; class: Reptilia ; order: Squamata ; family: Lacertidae ; genus: Darevskia ; scientificNameAuthorship: Mehely, 1909; **Location:** country: Georgia; stateProvince: Samtskhe-javakheti oblast; locality: Khanchkali lake (Zhdanovkani); decimalLatitude: 41.161321666667; decimalLongitude: 43.794278333333; geodeticDatum: WGS1984; georeferenceProtocol: GPS; **Identification:** identifiedBy: Arakelyan M.S.; **Event:** samplingProtocol: Captured by noose; eventDate: 2019-7-26; **Record Level:** language: en; rights: https://creativecommons.org/publicdomain/zero/1.0/; rightsHolder: Petrosyan V.G.; accessRights: http://vertnet.org/resources/norms.html; institutionCode: IEERASBIOINF; collectionCode: REPAMPHRU; basisOfRecord: HumanObservation
- **Type status:** Other material. **Occurrence:** catalogNumber: REPAMPHRU2019450; recordedBy: Osipov F.A.; individualCount: 1; sex: fmale; lifeStage: adult; occurrenceID: urn:IEERASBIOINF:REPAMPHRU2019450; **Taxon:** scientificName: Darevskia armeniaca; kingdom: Animalia ; phylum: Chordata ; class: Reptilia ; order: Squamata ; family: Lacertidae ; genus: Darevskia ; scientificNameAuthorship: Mehely, 1909; **Location:** country: Georgia; stateProvince: Samtskhe-Javakheti oblast; locality: Khanchali lake; decimalLatitude: 41.481283333333; decimalLongitude: 43.2802; geodeticDatum: WGS1984; georeferenceProtocol: GPS; **Identification:** identifiedBy: Arakelyan M.S.; **Event:** samplingProtocol: Captured by noose; eventDate: 2019-7-26; **Record Level:** language: en; rights: https://creativecommons.org/publicdomain/zero/1.0/; rightsHolder: Petrosyan V.G.; accessRights: http://vertnet.org/resources/norms.html; institutionCode: IEERASBIOINF; collectionCode: REPAMPHRU; basisOfRecord: HumanObservation
- **Type status:** Other material. **Occurrence:** catalogNumber: REPAMPHRU2019458; recordedBy: Osipov F.A.; individualCount: 7; sex: fmale; lifeStage: adult; occurrenceID: urn:IEERASBIOINF:REPAMPHRU2019458; **Taxon:** scientificName: Darevskia armeniaca; kingdom: Animalia ; phylum: Chordata ; class: Reptilia ; order: Squamata ; family: Lacertidae ; genus: Darevskia ; scientificNameAuthorship: Mehely, 1909; **Location:** country: Armenia; stateProvince: Kotayk Province; locality: Tsakhkadzor; decimalLatitude: 40.53515; decimalLongitude: 44.6972; geodeticDatum: WGS1984; georeferenceProtocol: GPS; **Identification:** identifiedBy: Arakelyan M.S.; **Event:** samplingProtocol: Captured by noose; eventDate: 2018-7-9; **Record Level:** language: en; rights: https://creativecommons.org/publicdomain/zero/1.0/; rightsHolder: Petrosyan V.G.; accessRights: http://vertnet.org/resources/norms.html; institutionCode: IEERASBIOINF; collectionCode: REPAMPHRU; basisOfRecord: HumanObservation
- **Type status:** Other material. **Occurrence:** catalogNumber: REPAMPHRU2019462; recordedBy: Osipov F.A.; individualCount: 1; sex: fmale; lifeStage: adult; occurrenceID: urn:IEERASBIOINF:REPAMPHRU2019462; **Taxon:** scientificName: Darevskia armeniaca; kingdom: Animalia ; phylum: Chordata ; class: Reptilia ; order: Squamata ; family: Lacertidae ; genus: Darevskia ; scientificNameAuthorship: Mehely, 1909; **Location:** country: Armenia; stateProvince: Kotayk Province; locality: Artavazd; decimalLatitude: 40.620316666667; decimalLongitude: 44.56305; geodeticDatum: WGS1984; georeferenceProtocol: GPS; **Identification:** identifiedBy: Arakelyan M.S.; **Event:** samplingProtocol: Captured by noose; eventDate: 2018-7-14; **Record Level:** language: en; rights: https://creativecommons.org/publicdomain/zero/1.0/; rightsHolder: Petrosyan V.G.; accessRights: http://vertnet.org/resources/norms.html; institutionCode: IEERASBIOINF; collectionCode: REPAMPHRU; basisOfRecord: HumanObservation
- **Type status:** Other material. **Occurrence:** catalogNumber: REPAMPHRU2019466; recordedBy: Osipov F.A.; individualCount: 1; sex: fmale; lifeStage: adult; occurrenceID: urn:IEERASBIOINF:REPAMPHRU2019466; **Taxon:** scientificName: Darevskia armeniaca; kingdom: Animalia ; phylum: Chordata ; class: Reptilia ; order: Squamata ; family: Lacertidae ; genus: Darevskia ; scientificNameAuthorship: Mehely, 1909; **Location:** country: Georgia; stateProvince: Samtskhe-javakheti oblast; locality: Akhalkalaki (Rio Kirkh-Bulakhi); decimalLatitude: 41.393743333333; decimalLongitude: 43.469711666667; geodeticDatum: WGS1984; georeferenceProtocol: GPS; **Identification:** identifiedBy: Arakelyan M.S.; **Event:** samplingProtocol: Captured by noose; eventDate: 2019-7-26; **Record Level:** language: en; rights: https://creativecommons.org/publicdomain/zero/1.0/; rightsHolder: Petrosyan V.G.; accessRights: http://vertnet.org/resources/norms.html; institutionCode: IEERASBIOINF; collectionCode: REPAMPHRU; basisOfRecord: HumanObservation
- **Type status:** Other material. **Occurrence:** catalogNumber: REPAMPHRU2019472; recordedBy: Osipov F.A.; individualCount: 1; sex: fmale; lifeStage: adult; occurrenceID: urn:IEERASBIOINF:REPAMPHRU2019472; **Taxon:** scientificName: Darevskia armeniaca; kingdom: Animalia ; phylum: Chordata ; class: Reptilia ; order: Squamata ; family: Lacertidae ; genus: Darevskia ; scientificNameAuthorship: Mehely, 1909; **Location:** country: Armenia; stateProvince: Lori Province; locality: Privolnoe; decimalLatitude: 41.148065; decimalLongitude: 44.466437; geodeticDatum: WGS1984; georeferenceProtocol: GPS; **Identification:** identifiedBy: Arakelyan M.S.; **Event:** samplingProtocol: Captured by noose; eventDate: 2019-6-18; **Record Level:** language: en; rights: https://creativecommons.org/publicdomain/zero/1.0/; rightsHolder: Petrosyan V.G.; accessRights: http://vertnet.org/resources/norms.html; institutionCode: IEERASBIOINF; collectionCode: REPAMPHRU; basisOfRecord: HumanObservation
- **Type status:** Other material. **Occurrence:** catalogNumber: REPAMPHRU2019474; recordedBy: Osipov F.A.; individualCount: 1; sex: fmale; lifeStage: adult; occurrenceID: urn:IEERASBIOINF:REPAMPHRU2019474; **Taxon:** scientificName: Darevskia armeniaca; kingdom: Animalia ; phylum: Chordata ; class: Reptilia ; order: Squamata ; family: Lacertidae ; genus: Darevskia ; scientificNameAuthorship: Mehely, 1909; **Location:** country: Armenia; stateProvince: Lori Province; locality: Dorbantvank; decimalLatitude: 41.113712; decimalLongitude: 44.435583; geodeticDatum: WGS1984; georeferenceProtocol: GPS; **Identification:** identifiedBy: Arakelyan M.S.; **Event:** samplingProtocol: Captured by noose; eventDate: 2019-6-19; **Record Level:** language: en; rights: https://creativecommons.org/publicdomain/zero/1.0/; rightsHolder: Petrosyan V.G.; accessRights: http://vertnet.org/resources/norms.html; institutionCode: IEERASBIOINF; collectionCode: REPAMPHRU; basisOfRecord: HumanObservation
- **Type status:** Other material. **Occurrence:** catalogNumber: REPAMPHRU2019476; recordedBy: Osipov F.A.; individualCount: 1; sex: fmale; lifeStage: adult; occurrenceID: urn:IEERASBIOINF:REPAMPHRU2019476; **Taxon:** scientificName: Darevskia armeniaca; kingdom: Animalia ; phylum: Chordata ; class: Reptilia ; order: Squamata ; family: Lacertidae ; genus: Darevskia ; scientificNameAuthorship: Mehely, 1909; **Location:** country: Armenia; stateProvince: Lori Province; locality: Tashir; decimalLatitude: 41.154566; decimalLongitude: 44.308898; geodeticDatum: WGS1984; georeferenceProtocol: GPS; **Identification:** identifiedBy: Arakelyan M.S.; **Event:** samplingProtocol: Captured by noose; eventDate: 2019-6-19; **Record Level:** language: en; rights: https://creativecommons.org/publicdomain/zero/1.0/; rightsHolder: Petrosyan V.G.; accessRights: http://vertnet.org/resources/norms.html; institutionCode: IEERASBIOINF; collectionCode: REPAMPHRU; basisOfRecord: HumanObservation

#### Notes

**Figure 3.**
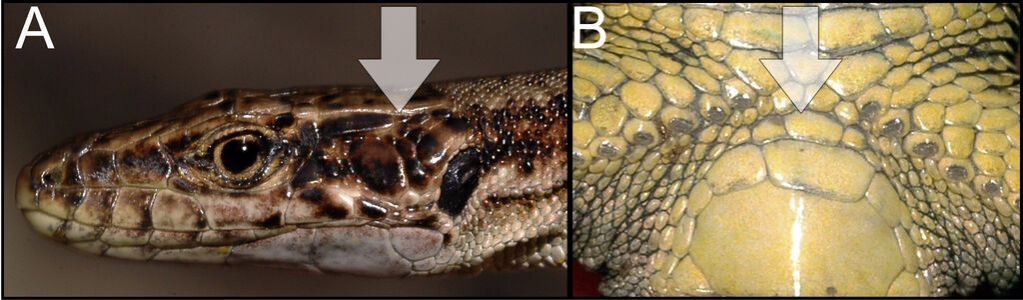
Distinguishing characters of *D. armeniaca*. **A.** Between the central temporal and tympanum shields are two scales of similar sizes or the central temporal shield touches the tympanic scale (indicated by arrow). **B.** In front of the large anal shield, there are one or two enlarged pre-anal scales of different size than the other pre-anals (indicated by arrow). Photos by I. Kropachev.

Parthenogenetic species, *D. armeniaca*, originated as a result of the interspecific hybridisation between bisexual species *D. valentini* (“paternal”) and *D. mixta* (“maternal”) (Darevsky 1967, Murphy et al. 2000). However, there is a hypothesis that *D. armeniaca* might be a descendant of the hybridisation between *D. valentini* males and parthenogenetic *D. dahli* (Tarkhnishvili et al. 2017). *Darevskia armeniaca* identification from different SZs was performed in different years by different authors using allozyme loci, mt-DNA, multilocus DNA fingerprinting, mini- and micro-satellite markers and morphological features (Darevsky 1966, Darevsky 1967, Darevsky and Danielyan 1968, Uzzell and Darevsky 1975, Tokarskaya et al. 2001, Malysheva et al. 2007a, Malysheva et al. 2007b, Martirosian et al. 2003, Petrosyan et al. 2003). In the field, *D. armeniaca* was identified using species identification guides (Darevsky 1967, Arakelyan et al. 2011) (Fig. 3), since it has previously been shown that identification, based on visual observation, did not cause confusion between species (Petrosyan et al. 2019a, Petrosyan et al. 2019b). During our field surveys, we captured and identified 42 individuals in 24 SZs. All individuals were found to be females, determined by visual inspection of the genitals.

*Darevskia armeniaca* is widespread in the Transcaucasus in the north-west of Armenia, in the western part of Azerbaijan, in the south of Georgia and in the north-eastern part of Turkey (Petrosyan et al. 2019a, Petrosyan et al. 2019b). Species were identified in 24 (20%) SZs of 121 examined sites. In the study region, eight zones of co-existence with the “paternal” species *D. valentini* were revealed. The numbers of SZs, where there was сo-existence with other parthenogenetic species *D. dahli*, *D. unisexualis* and *D. rostombekowi*, were eleven, four and five, respectively. Hybrid individuals of *D. valentini* x *D. armeniaca* were found in three SZs (Lchashen, Kuchak and Tezh), which were previously stated in literature (Danielyan et al. 2008, Carretero et al. 2018). Our data showed that the identified SZs are located in diverse habitats, such as outcrops of rocks, large stones and clay rocks in mountain steppes, mountain meadows, mountain forests and urbanised biotopes of central and eastern parts of Armenia and southern Georgia (Fig. 1).

### Darevskia dahli

(Darevsky, 1957)

57D2FDD3-420C-5608-BE04-3C6E8E347CC8

#### Materials

- **Type status:** Other material. **Occurrence:** catalogNumber: REPAMPHRU2018392; recordedBy: Osipov F.A.; individualCount: 1; sex: fmale; lifeStage: adult; occurrenceID: urn:IEERASBIOINF:REPAMPHRU2018392; **Taxon:** scientificName: Darevskia dahli; kingdom: Animalia ; phylum: Chordata ; class: Reptilia ; order: Squamata ; family: Lacertidae ; genus: Darevskia ; scientificNameAuthorship: Darevsky, 1957; **Location:** country: Armenia; stateProvince: Tavush Province; locality: Dilijan; decimalLatitude: 40.733998; decimalLongitude: 44.81778; geodeticDatum: WGS1984; georeferenceProtocol: GPS; **Identification:** identifiedBy: Arakelyan M.S.; **Event:** samplingProtocol: Captured by noose; eventDate: 2018-7-9; **Record Level:** language: en; rights: https://creativecommons.org/publicdomain/zero/1.0/; rightsHolder: Petrosyan V.G.; accessRights: http://vertnet.org/resources/norms.html; institutionCode: IEERASBIOINF; collectionCode: REPAMPHRU; basisOfRecord: HumanObservation
- **Type status:** Other material. **Occurrence:** catalogNumber: REPAMPHRU2018414; recordedBy: Osipov F.A.; individualCount: 2; sex: fmale; lifeStage: adult; occurrenceID: urn:IEERASBIOINF:REPAMPHRU2018414; **Taxon:** scientificName: Darevskia dahli; kingdom: Animalia ; phylum: Chordata ; class: Reptilia ; order: Squamata ; family: Lacertidae ; genus: Darevskia ; scientificNameAuthorship: Darevsky, 1957; **Location:** country: Armenia; stateProvince: Lori Province; locality: Gogaran; decimalLatitude: 40.895442; decimalLongitude: 44.202444; geodeticDatum: WGS1984; georeferenceProtocol: GPS; **Identification:** identifiedBy: Arakelyan M.S.; **Event:** samplingProtocol: Captured by noose; eventDate: 2018-7-16; **Record Level:** language: en; rights: https://creativecommons.org/publicdomain/zero/1.0/; rightsHolder: Petrosyan V.G.; accessRights: http://vertnet.org/resources/norms.html; institutionCode: IEERASBIOINF; collectionCode: REPAMPHRU; basisOfRecord: HumanObservation
- **Type status:** Other material. **Occurrence:** catalogNumber: REPAMPHRU2019418; recordedBy: Osipov F.A.; individualCount: 1; sex: fmale; lifeStage: adult; occurrenceID: urn:IEERASBIOINF:REPAMPHRU2019418; **Taxon:** scientificName: Darevskia dahli; kingdom: Animalia ; phylum: Chordata ; class: Reptilia ; order: Squamata ; family: Lacertidae ; genus: Darevskia ; scientificNameAuthorship: Darevsky, 1957; **Location:** country: Armenia; stateProvince: Aragatsotn Province; locality: Tsilkar; decimalLatitude: 40.736893; decimalLongitude: 44.197427; geodeticDatum: WGS1984; georeferenceProtocol: GPS; **Identification:** identifiedBy: Arakelyan M.S.; **Event:** samplingProtocol: Captured by noose; eventDate: 2019-7-16; **Record Level:** language: en; rights: https://creativecommons.org/publicdomain/zero/1.0/; rightsHolder: Petrosyan V.G.; accessRights: http://vertnet.org/resources/norms.html; institutionCode: IEERASBIOINF; collectionCode: REPAMPHRU; basisOfRecord: HumanObservation
- **Type status:** Other material. **Occurrence:** catalogNumber: REPAMPHRU2019420; recordedBy: Osipov F.A.; individualCount: 5; sex: fmale; lifeStage: adult; occurrenceID: urn:IEERASBIOINF:REPAMPHRU2019420; **Taxon:** scientificName: Darevskia dahli; kingdom: Animalia ; phylum: Chordata ; class: Reptilia ; order: Squamata ; family: Lacertidae ; genus: Darevskia ; scientificNameAuthorship: Darevsky, 1957; **Location:** country: Armenia; stateProvince: Lori Province; locality: Pushkin pass; decimalLatitude: 40.917347; decimalLongitude: 44.436738; geodeticDatum: WGS1984; georeferenceProtocol: GPS; **Identification:** identifiedBy: Arakelyan M.S.; **Event:** samplingProtocol: Captured by noose; eventDate: 2019-7-16; **Record Level:** language: en; rights: https://creativecommons.org/publicdomain/zero/1.0/; rightsHolder: Petrosyan V.G.; accessRights: http://vertnet.org/resources/norms.html; institutionCode: IEERASBIOINF; collectionCode: REPAMPHRU; basisOfRecord: HumanObservation
- **Type status:** Other material. **Occurrence:** catalogNumber: REPAMPHRU2019422; recordedBy: Osipov F.A.; individualCount: 1; sex: fmale; lifeStage: adult; occurrenceID: urn:IEERASBIOINF:REPAMPHRU2019422; **Taxon:** scientificName: Darevskia dahli; kingdom: Animalia ; phylum: Chordata ; class: Reptilia ; order: Squamata ; family: Lacertidae ; genus: Darevskia ; scientificNameAuthorship: Darevsky, 1957; **Location:** country: Armenia; stateProvince: Lori Province; locality: Pushkin memorial end; decimalLatitude: 40.93278; decimalLongitude: 44.44017; geodeticDatum: WGS1984; georeferenceProtocol: GPS; **Identification:** identifiedBy: Arakelyan M.S.; **Event:** samplingProtocol: Captured by noose; eventDate: 2019-7-16; **Record Level:** language: en; rights: https://creativecommons.org/publicdomain/zero/1.0/; rightsHolder: Petrosyan V.G.; accessRights: http://vertnet.org/resources/norms.html; institutionCode: IEERASBIOINF; collectionCode: REPAMPHRU; basisOfRecord: HumanObservation
- **Type status:** Other material. **Occurrence:** catalogNumber: REPAMPHRU2019424; recordedBy: Osipov F.A.; individualCount: 2; sex: fmale; lifeStage: adult; occurrenceID: urn:IEERASBIOINF:REPAMPHRU2019424; **Taxon:** scientificName: Darevskia dahli; kingdom: Animalia ; phylum: Chordata ; class: Reptilia ; order: Squamata ; family: Lacertidae ; genus: Darevskia ; scientificNameAuthorship: Darevsky, 1957; **Location:** country: Armenia; stateProvince: Lori Province; locality: Dendropark; decimalLatitude: 40.93852; decimalLongitude: 44.479332; geodeticDatum: WGS1984; georeferenceProtocol: GPS; **Identification:** identifiedBy: Arakelyan M.S.; **Event:** samplingProtocol: Captured by noose; eventDate: 2019-7-16; **Record Level:** language: en; rights: https://creativecommons.org/publicdomain/zero/1.0/; rightsHolder: Petrosyan V.G.; accessRights: http://vertnet.org/resources/norms.html; institutionCode: IEERASBIOINF; collectionCode: REPAMPHRU; basisOfRecord: HumanObservation
- **Type status:** Other material. **Occurrence:** catalogNumber: REPAMPHRU2019426; recordedBy: Osipov F.A.; individualCount: 3; sex: fmale; lifeStage: adult; occurrenceID: urn:IEERASBIOINF:REPAMPHRU2019426; **Taxon:** scientificName: Darevskia dahli; kingdom: Animalia ; phylum: Chordata ; class: Reptilia ; order: Squamata ; family: Lacertidae ; genus: Darevskia ; scientificNameAuthorship: Darevsky, 1957; **Location:** country: Armenia; stateProvince: Lori Province; locality: Dzoraget; decimalLatitude: 41.014219; decimalLongitude: 44.379631; geodeticDatum: WGS1984; georeferenceProtocol: GPS; **Identification:** identifiedBy: Arakelyan M.S.; **Event:** samplingProtocol: Captured by noose; eventDate: 2019-7-16; **Record Level:** language: en; rights: https://creativecommons.org/publicdomain/zero/1.0/; rightsHolder: Petrosyan V.G.; accessRights: http://vertnet.org/resources/norms.html; institutionCode: IEERASBIOINF; collectionCode: REPAMPHRU; basisOfRecord: HumanObservation
- **Type status:** Other material. **Occurrence:** catalogNumber: REPAMPHRU2019434; recordedBy: Osipov F.A.; individualCount: 1; sex: fmale; lifeStage: adult; occurrenceID: urn:IEERASBIOINF:REPAMPHRU2019434; **Taxon:** scientificName: Darevskia dahli; kingdom: Animalia ; phylum: Chordata ; class: Reptilia ; order: Squamata ; family: Lacertidae ; genus: Darevskia ; scientificNameAuthorship: Darevsky, 1957; **Location:** country: Armenia; stateProvince: Tavush Province; locality: Serpentine from Ijevan; decimalLatitude: 40.868307; decimalLongitude: 45.187475; geodeticDatum: WGS1984; georeferenceProtocol: GPS; **Identification:** identifiedBy: Arakelyan M.S.; **Event:** samplingProtocol: Captured by noose; eventDate: 2019-7-22; **Record Level:** language: en; rights: https://creativecommons.org/publicdomain/zero/1.0/; rightsHolder: Petrosyan V.G.; accessRights: http://vertnet.org/resources/norms.html; institutionCode: IEERASBIOINF; collectionCode: REPAMPHRU; basisOfRecord: HumanObservation
- **Type status:** Other material. **Occurrence:** catalogNumber: REPAMPHRU2019437; recordedBy: Osipov F.A.; individualCount: 1; sex: fmale; lifeStage: adult; occurrenceID: urn:IEERASBIOINF:REPAMPHRU2019437; **Taxon:** scientificName: Darevskia dahli; kingdom: Animalia ; phylum: Chordata ; class: Reptilia ; order: Squamata ; family: Lacertidae ; genus: Darevskia ; scientificNameAuthorship: Darevsky, 1957; **Location:** country: Armenia; stateProvince: Lori Province; locality: Fioletova; decimalLatitude: 40.715555; decimalLongitude: 44.77062; geodeticDatum: WGS1984; georeferenceProtocol: GPS; **Identification:** identifiedBy: Arakelyan M.S.; **Event:** samplingProtocol: Captured by noose; eventDate: 2019-7-24; **Record Level:** language: en; rights: https://creativecommons.org/publicdomain/zero/1.0/; rightsHolder: Petrosyan V.G.; accessRights: http://vertnet.org/resources/norms.html; institutionCode: IEERASBIOINF; collectionCode: REPAMPHRU; basisOfRecord: HumanObservation
- **Type status:** Other material. **Occurrence:** catalogNumber: REPAMPHRU2019440; recordedBy: Osipov F.A.; individualCount: 7; sex: fmale; lifeStage: adult; occurrenceID: urn:IEERASBIOINF:REPAMPHRU2019440; **Taxon:** scientificName: Darevskia dahli; kingdom: Animalia ; phylum: Chordata ; class: Reptilia ; order: Squamata ; family: Lacertidae ; genus: Darevskia ; scientificNameAuthorship: Darevsky, 1957; **Location:** country: Armenia; stateProvince: Shirak Province; locality: Keti; decimalLatitude: 40.864013; decimalLongitude: 43.841847; geodeticDatum: WGS1984; georeferenceProtocol: GPS; **Identification:** identifiedBy: Arakelyan M.S.; **Event:** samplingProtocol: Captured by noose; eventDate: 2019-7-25; **Record Level:** language: en; rights: https://creativecommons.org/publicdomain/zero/1.0/; rightsHolder: Petrosyan V.G.; accessRights: http://vertnet.org/resources/norms.html; institutionCode: IEERASBIOINF; collectionCode: REPAMPHRU; basisOfRecord: HumanObservation
- **Type status:** Other material. **Occurrence:** catalogNumber: REPAMPHRU2019455; recordedBy: Osipov F.A.; individualCount: 1; sex: fmale; lifeStage: adult; occurrenceID: urn:IEERASBIOINF:REPAMPHRU2019455; **Taxon:** scientificName: Darevskia dahli; kingdom: Animalia ; phylum: Chordata ; class: Reptilia ; order: Squamata ; family: Lacertidae ; genus: Darevskia ; scientificNameAuthorship: Darevsky, 1957; **Location:** country: Armenia; stateProvince: Tavush Province; locality: Forest area near Acharkut; decimalLatitude: 41.026279; decimalLongitude: 45.052574; geodeticDatum: WGS1984; georeferenceProtocol: GPS; **Identification:** identifiedBy: Arakelyan M.S.; **Event:** samplingProtocol: Captured by noose; eventDate: 2018-7-23; **Record Level:** language: en; rights: https://creativecommons.org/publicdomain/zero/1.0/; rightsHolder: Petrosyan V.G.; accessRights: http://vertnet.org/resources/norms.html; institutionCode: IEERASBIOINF; collectionCode: REPAMPHRU; basisOfRecord: HumanObservation
- **Type status:** Other material. **Occurrence:** catalogNumber: REPAMPHRU2019468; recordedBy: Osipov F.A.; individualCount: 1; sex: fmale; lifeStage: adult; occurrenceID: urn:IEERASBIOINF:REPAMPHRU2019468; **Taxon:** scientificName: Darevskia dahli; kingdom: Animalia ; phylum: Chordata ; class: Reptilia ; order: Squamata ; family: Lacertidae ; genus: Darevskia ; scientificNameAuthorship: Darevsky, 1957; **Location:** country: Georgia; stateProvince: Kvemo Kartli region; locality: Guguti; decimalLatitude: 41.200509; decimalLongitude: 44.552717; geodeticDatum: WGS1984; georeferenceProtocol: GPS; **Identification:** identifiedBy: Arakelyan M.S.; **Event:** samplingProtocol: Captured by noose; eventDate: 2019-6-18; **Record Level:** language: en; rights: https://creativecommons.org/publicdomain/zero/1.0/; rightsHolder: Petrosyan V.G.; accessRights: http://vertnet.org/resources/norms.html; institutionCode: IEERASBIOINF; collectionCode: REPAMPHRU; basisOfRecord: HumanObservation
- **Type status:** Other material. **Occurrence:** catalogNumber: REPAMPHRU2019470; recordedBy: Osipov F.A.; individualCount: 1; sex: fmale; lifeStage: adult; occurrenceID: urn:IEERASBIOINF:REPAMPHRU2019470; **Taxon:** scientificName: Darevskia dahli; kingdom: Animalia ; phylum: Chordata ; class: Reptilia ; order: Squamata ; family: Lacertidae ; genus: Darevskia ; scientificNameAuthorship: Darevsky, 1957; **Location:** country: Armenia; stateProvince: Lori Province; locality: Karmir Ageg; decimalLatitude: 40.97993; decimalLongitude: 44.56121; geodeticDatum: WGS1984; georeferenceProtocol: GPS; **Identification:** identifiedBy: Arakelyan M.S.; **Event:** samplingProtocol: Captured by noose; eventDate: 2019-6-18; **Record Level:** language: en; rights: https://creativecommons.org/publicdomain/zero/1.0/; rightsHolder: Petrosyan V.G.; accessRights: http://vertnet.org/resources/norms.html; institutionCode: IEERASBIOINF; collectionCode: REPAMPHRU; basisOfRecord: HumanObservation
- **Type status:** Other material. **Occurrence:** catalogNumber: REPAMPHRU2019473; recordedBy: Osipov F.A.; individualCount: 1; sex: fmale; lifeStage: adult; occurrenceID: urn:IEERASBIOINF:REPAMPHRU2019473; **Taxon:** scientificName: Darevskia dahli; kingdom: Animalia ; phylum: Chordata ; class: Reptilia ; order: Squamata ; family: Lacertidae ; genus: Darevskia ; scientificNameAuthorship: Darevsky, 1957; **Location:** country: Armenia; stateProvince: Lori Province; locality: Dorbantvank; decimalLatitude: 41.113712; decimalLongitude: 44.435583; geodeticDatum: WGS1984; georeferenceProtocol: GPS; **Identification:** identifiedBy: Arakelyan M.S.; **Event:** samplingProtocol: Captured by noose; eventDate: 2019-6-19; **Record Level:** language: en; rights: https://creativecommons.org/publicdomain/zero/1.0/; rightsHolder: Petrosyan V.G.; accessRights: http://vertnet.org/resources/norms.html; institutionCode: IEERASBIOINF; collectionCode: REPAMPHRU; basisOfRecord: HumanObservation
- **Type status:** Other material. **Occurrence:** catalogNumber: REPAMPHRU2019475; recordedBy: Osipov F.A.; individualCount: 1; sex: fmale; lifeStage: adult; occurrenceID: urn:IEERASBIOINF:REPAMPHRU2019475; **Taxon:** scientificName: Darevskia dahli; kingdom: Animalia ; phylum: Chordata ; class: Reptilia ; order: Squamata ; family: Lacertidae ; genus: Darevskia ; scientificNameAuthorship: Darevsky, 1957; **Location:** country: Armenia; stateProvince: Lori Province; locality: Tashir; decimalLatitude: 41.154566; decimalLongitude: 44.308898; geodeticDatum: WGS1984; georeferenceProtocol: GPS; **Identification:** identifiedBy: Arakelyan M.S.; **Event:** samplingProtocol: Captured by noose; eventDate: 2019-6-19; **Record Level:** language: en; rights: https://creativecommons.org/publicdomain/zero/1.0/; rightsHolder: Petrosyan V.G.; accessRights: http://vertnet.org/resources/norms.html; institutionCode: IEERASBIOINF; collectionCode: REPAMPHRU; basisOfRecord: HumanObservation

#### Notes

**Figure 4.**
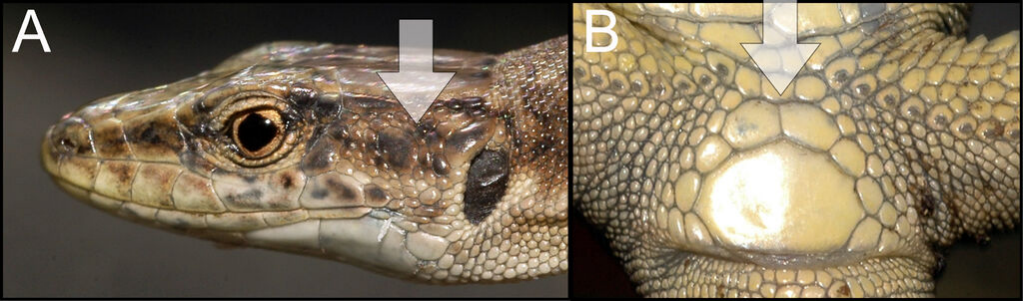
Distinguishing characters of *D. dahli*. **A.** Between the central temporal and tympanum shields in the narrowest place in the same row, there are 2-3 enlarged shields (indicated by arrow); **B.** In front of the large anal shield, there are always two symmetrical enlarged pre-anal ones (indicated by arrow). The differences between *D. dahli* and *D. armeniaca* also exist in number and placement of ciliar granules. Photos by I. Kropachev.

The parthenogenetic *D. dahli* lizard is of hybrid origin (Darevsky 1967, Uzzell and Darevsky 1975). The "maternal" species of *D. dahli* is *D. mixta*; "paternal" is *D. portschinskii* (Darevsky 1967, Uzzell and Darevsky 1975, Moritz et al. 1992, Fu et al. 1999, Murphy et al. 2000). Species identification of *D. dahli* from SZs was carried out by authors in different years using allozyme loci, mt-DNA, multilocus DNA fingerprinting, mini- and micro-satellite markers and morphological features (Darevsky 1966, Darevsky 1967, Uzzell and Darevsky 1975, Fu et al. 1999, Ryabinina et al. 1999, Murphy et al. 2000, Tokarskaya et al. 2001, Davoyan et al. 2007, Vergun et al. 2014, Arakelyan et al. 2011). In the field, *D. dahli* was identified using species identification guides (Darevsky 1967), (Fig. 4), since it has previously been shown that identification, based on visual observation, did not cause confusion between the species (Tarkhnishvili et al. 2010, Arakelyan et al. 2011). During our field survey, we captured and identified 29 individuals. All individuals were found to be females, determined by visual inspection of the genitals.

*Darevskia dahli* is widespread in north-eastern Armenia, western Azerbaijan and southern and central Georgia. Suitable habitats of the species in north-eastern Armenia are divided into seven vast isolated areas assigned to highland forest, meadow and steppe zones (Arakelyan et al. 2011, Petrosyan et al. 2020). During the survey, 15 SZs have been identified, 12 of which being located in the two north-western Provinces of Tavush and Lori (Arakelyan et al. 2011). *Darevskia dahli* co-exist*s* in three SZs with a "paternal" species *D. portschinskii* and with *D. armeniaca*, *D. rostombekowi* and *D. unisexualis* in eleven, three and two SZs, respectively. Identified SZs are located in various biotopes: mountain forests, mountain meadows, mountain steppes and urbanised biotopes.

### Darevskia rostombekowi

(Darevsky, 1957)

79DD99D5-1902-5EEF-A9FA-AF472B5A7C93

#### Materials

- **Type status:** Other material. **Occurrence:** catalogNumber: REPAMPHRU2018394; recordedBy: Osipov F.A.; individualCount: 1; sex: fmale; lifeStage: adult; occurrenceID: urn:IEERASBIOINF:REPAMPHRU2018394; **Taxon:** scientificName: Darevskia rostombekowi; kingdom: Animalia ; phylum: Chordata ; class: Reptilia ; order: Squamata ; family: Lacertidae ; genus: Darevskia ; scientificNameAuthorship: Darevsky, 1957; **Location:** country: Armenia; stateProvince: Tavush Province; locality: Dilijan; decimalLatitude: 40.733998; decimalLongitude: 44.81778; geodeticDatum: WGS1984; georeferenceProtocol: GPS; **Identification:** identifiedBy: Arakelyan M.S.; **Event:** samplingProtocol: Captured by noose; eventDate: 2018-7-9; **Record Level:** language: en; rights: https://creativecommons.org/publicdomain/zero/1.0/; rightsHolder: Petrosyan V.G.; accessRights: http://vertnet.org/resources/norms.html; institutionCode: IEERASBIOINF; collectionCode: REPAMPHRU; basisOfRecord: HumanObservation
- **Type status:** Other material. **Occurrence:** catalogNumber: REPAMPHRU2018395; recordedBy: Osipov F.A.; individualCount: 1; sex: fmale; lifeStage: adult; occurrenceID: urn:IEERASBIOINF:REPAMPHRU2018395; **Taxon:** scientificName: Darevskia rostombekowi; kingdom: Animalia ; phylum: Chordata ; class: Reptilia ; order: Squamata ; family: Lacertidae ; genus: Darevskia ; scientificNameAuthorship: Darevsky, 1957; **Location:** country: Armenia; stateProvince: Tavush Province; locality: Road to Dilijan - Hagarcin; decimalLatitude: 40.76671; decimalLongitude: 44.919407; geodeticDatum: WGS1984; georeferenceProtocol: GPS; **Identification:** identifiedBy: Arakelyan M.S.; **Event:** samplingProtocol: Captured by noose; eventDate: 2018-7-9; **Record Level:** language: en; rights: https://creativecommons.org/publicdomain/zero/1.0/; rightsHolder: Petrosyan V.G.; accessRights: http://vertnet.org/resources/norms.html; institutionCode: IEERASBIOINF; collectionCode: REPAMPHRU; basisOfRecord: HumanObservation
- **Type status:** Other material. **Occurrence:** catalogNumber: REPAMPHRU2018399; recordedBy: Osipov F.A.; individualCount: 2; sex: fmale; lifeStage: adult; occurrenceID: urn:IEERASBIOINF:REPAMPHRU2018399; **Taxon:** scientificName: Darevskia rostombekowi; kingdom: Animalia ; phylum: Chordata ; class: Reptilia ; order: Squamata ; family: Lacertidae ; genus: Darevskia ; scientificNameAuthorship: Darevsky, 1957; **Location:** country: Armenia; stateProvince: Tavush Province; locality: Haghartsin Monastery; decimalLatitude: 40.801931; decimalLongitude: 44.890573; geodeticDatum: WGS1984; georeferenceProtocol: GPS; **Identification:** identifiedBy: Arakelyan M.S.; **Event:** samplingProtocol: Captured by noose; eventDate: 2018-7-9; **Record Level:** language: en; rights: https://creativecommons.org/publicdomain/zero/1.0/; rightsHolder: Petrosyan V.G.; accessRights: http://vertnet.org/resources/norms.html; institutionCode: IEERASBIOINF; collectionCode: REPAMPHRU; basisOfRecord: HumanObservation
- **Type status:** Other material. **Occurrence:** catalogNumber: REPAMPHRU2018404; recordedBy: Osipov F.A.; individualCount: 1; sex: fmale; lifeStage: adult; occurrenceID: urn:IEERASBIOINF:REPAMPHRU2018404; **Taxon:** scientificName: Darevskia rostombekowi; kingdom: Animalia ; phylum: Chordata ; class: Reptilia ; order: Squamata ; family: Lacertidae ; genus: Darevskia ; scientificNameAuthorship: Darevsky, 1957; **Location:** country: Armenia; stateProvince: Gegharkunik Province; locality: Tsovak; decimalLatitude: 40.185681; decimalLongitude: 45.623972; geodeticDatum: WGS1984; georeferenceProtocol: GPS; **Identification:** identifiedBy: Arakelyan M.S.; **Event:** samplingProtocol: Captured by noose; eventDate: 2018-7-10; **Record Level:** language: en; rights: https://creativecommons.org/publicdomain/zero/1.0/; rightsHolder: Petrosyan V.G.; accessRights: http://vertnet.org/resources/norms.html; institutionCode: IEERASBIOINF; collectionCode: REPAMPHRU; basisOfRecord: HumanObservation
- **Type status:** Other material. **Occurrence:** catalogNumber: REPAMPHRU2019433; recordedBy: Osipov F.A.; individualCount: 1; sex: fmale; lifeStage: adult; occurrenceID: urn:IEERASBIOINF:REPAMPHRU2019433; **Taxon:** scientificName: Darevskia rostombekowi; kingdom: Animalia ; phylum: Chordata ; class: Reptilia ; order: Squamata ; family: Lacertidae ; genus: Darevskia ; scientificNameAuthorship: Darevsky, 1957; **Location:** country: Armenia; stateProvince: Tavush Province; locality: Serpentine from Ijevan; decimalLatitude: 40.868307; decimalLongitude: 45.187475; geodeticDatum: WGS1984; georeferenceProtocol: GPS; **Identification:** identifiedBy: Arakelyan M.S.; **Event:** samplingProtocol: Captured by noose; eventDate: 2019-7-22; **Record Level:** language: en; rights: https://creativecommons.org/publicdomain/zero/1.0/; rightsHolder: Petrosyan V.G.; accessRights: http://vertnet.org/resources/norms.html; institutionCode: IEERASBIOINF; collectionCode: REPAMPHRU; basisOfRecord: HumanObservation
- **Type status:** Other material. **Occurrence:** catalogNumber: REPAMPHRU2019435; recordedBy: Osipov F.A.; individualCount: 1; sex: fmale; lifeStage: adult; occurrenceID: urn:IEERASBIOINF:REPAMPHRU2019435; **Taxon:** scientificName: Darevskia rostombekowi; kingdom: Animalia ; phylum: Chordata ; class: Reptilia ; order: Squamata ; family: Lacertidae ; genus: Darevskia ; scientificNameAuthorship: Darevsky, 1957; **Location:** country: Armenia; stateProvince: Tavush Province; locality: Forest area near Acharkut; decimalLatitude: 41.026279; decimalLongitude: 45.052574; geodeticDatum: WGS1984; georeferenceProtocol: GPS; **Identification:** identifiedBy: Arakelyan M.S.; **Event:** samplingProtocol: Captured by noose; eventDate: 2019-7-23; **Record Level:** language: en; rights: https://creativecommons.org/publicdomain/zero/1.0/; rightsHolder: Petrosyan V.G.; accessRights: http://vertnet.org/resources/norms.html; institutionCode: IEERASBIOINF; collectionCode: REPAMPHRU; basisOfRecord: HumanObservation
- **Type status:** Other material. **Occurrence:** catalogNumber: REPAMPHRU2019439; recordedBy: Osipov F.A.; individualCount: 4; sex: fmale; lifeStage: adult; occurrenceID: urn:IEERASBIOINF:REPAMPHRU2019439; **Taxon:** scientificName: Darevskia rostombekowi; kingdom: Animalia ; phylum: Chordata ; class: Reptilia ; order: Squamata ; family: Lacertidae ; genus: Darevskia ; scientificNameAuthorship: Darevsky, 1957; **Location:** country: Armenia; stateProvince: Lori Province; locality: Fioletova; decimalLatitude: 40.715555; decimalLongitude: 44.77062; geodeticDatum: WGS1984; georeferenceProtocol: GPS; **Identification:** identifiedBy: Arakelyan M.S.; **Event:** samplingProtocol: Captured by noose; eventDate: 2019-7-24; **Record Level:** language: en; rights: https://creativecommons.org/publicdomain/zero/1.0/; rightsHolder: Petrosyan V.G.; accessRights: http://vertnet.org/resources/norms.html; institutionCode: IEERASBIOINF; collectionCode: REPAMPHRU; basisOfRecord: HumanObservation
- **Type status:** Other material. **Occurrence:** catalogNumber: REPAMPHRU2019446; recordedBy: Osipov F.A.; individualCount: 3; sex: fmale; lifeStage: adult; occurrenceID: urn:IEERASBIOINF:REPAMPHRU2019446; **Taxon:** scientificName: Darevskia rostombekowi; kingdom: Animalia ; phylum: Chordata ; class: Reptilia ; order: Squamata ; family: Lacertidae ; genus: Darevskia ; scientificNameAuthorship: Darevsky, 1957; **Location:** country: Armenia; stateProvince: Tavush Province; locality: Dilidjan forest; decimalLatitude: 40.757211666667; decimalLongitude: 44.804148333333; geodeticDatum: WGS1984; georeferenceProtocol: GPS; **Identification:** identifiedBy: Arakelyan M.S.; **Event:** samplingProtocol: Captured by noose; eventDate: 2019-7-22; **Record Level:** language: en; rights: https://creativecommons.org/publicdomain/zero/1.0/; rightsHolder: Petrosyan V.G.; accessRights: http://vertnet.org/resources/norms.html; institutionCode: IEERASBIOINF; collectionCode: REPAMPHRU; basisOfRecord: HumanObservation
- **Type status:** Other material. **Occurrence:** catalogNumber: REPAMPHRU2019457; recordedBy: Osipov F.A.; individualCount: 2; sex: fmale; lifeStage: adult; occurrenceID: urn:IEERASBIOINF:REPAMPHRU2019457; **Taxon:** scientificName: Darevskia rostombekowi; kingdom: Animalia ; phylum: Chordata ; class: Reptilia ; order: Squamata ; family: Lacertidae ; genus: Darevskia ; scientificNameAuthorship: Darevsky, 1957; **Location:** country: Armenia; stateProvince: Tavush Province; locality: Goshavank monastery; decimalLatitude: 40.729851666667; decimalLongitude: 44.99711; geodeticDatum: WGS1984; georeferenceProtocol: GPS; **Identification:** identifiedBy: Arakelyan M.S.; **Event:** samplingProtocol: Captured by noose; eventDate: 2018-7-21; **Record Level:** language: en; rights: https://creativecommons.org/publicdomain/zero/1.0/; rightsHolder: Petrosyan V.G.; accessRights: http://vertnet.org/resources/norms.html; institutionCode: IEERASBIOINF; collectionCode: REPAMPHRU; basisOfRecord: HumanObservation
- **Type status:** Other material. **Occurrence:** catalogNumber: REPAMPHRU2019480; recordedBy: Osipov F.A.; individualCount: 1; sex: fmale; lifeStage: adult; occurrenceID: urn:IEERASBIOINF:REPAMPHRU2019480; **Taxon:** scientificName: Darevskia rostombekowi; kingdom: Animalia ; phylum: Chordata ; class: Reptilia ; order: Squamata ; family: Lacertidae ; genus: Darevskia ; scientificNameAuthorship: Darevsky, 1957; **Location:** country: Armenia; stateProvince: Lori Province; locality: Spitak; decimalLatitude: 40.8249; decimalLongitude: 44.2775; geodeticDatum: WGS1984; georeferenceProtocol: GPS; **Identification:** identifiedBy: Arakelyan M.S.; **Event:** samplingProtocol: Captured by noose; eventDate: 2019-6-20; **Record Level:** language: en; rights: https://creativecommons.org/publicdomain/zero/1.0/; rightsHolder: Petrosyan V.G.; accessRights: http://vertnet.org/resources/norms.html; institutionCode: IEERASBIOINF; collectionCode: REPAMPHRU; basisOfRecord: HumanObservation

#### Notes

**Figure 5.**
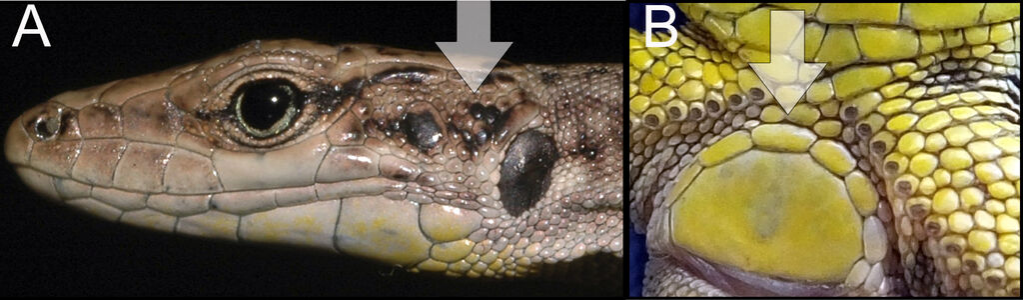
Distinguishing characters of *D. rostombekowi*. **A.** The central temporal shield is large, often divided into two; from the first upper temporal shield in the narrowest place, it is separated by 1-3 and, from the tympanum, by 2-4 transverse rows of enlarged shields (indicated by arrow); **B.** The anal shield is large, in front of it are symmetrically located four small and approximately equal in size pre-anal shields, the middle of which can be slightly increased (indicated by arrow). Photos by M. Arakelyan.

The parthenogenetic lizard *D. rostombekowi* has a hybrid origin (Darevsky 1967, Uzzell and Darevsky 1975). The "maternal" species for *D. rostombekowi* is *D. r. raddei* and "paternal" is *D. portschinskii* (Darevsky 1967, Uzzell and Darevsky 1975, Murphy et al. 2000, Ryskov et al. 2017). The identification of *Darevskia rostombekowi* in SZs was undertaken in different years using allozyme loci, mt-DNA, multilocus DNA fingerprinting, mini- and micro-satellite markers and morphological features (Darevsky 1967, Uzzell and Darevsky 1975, Macculloch et al. 1997, Fu et al. 2000a, Murphy et al. 2000, Martirosian et al. 2002, Petrosyan et al. 2003, Arakelyan et al. 2011, Ryskov et al. 2017). In the field, *D. rostombekowi* was identified using species identification guides according to Darevsky (Darevsky 1967) (Fig. 5), since it has previously been shown that identification, based on visual observation, did not cause confusion between the species (Martirosian et al. 2002, Petrosyan et al. 2003, Arakelyan et al. 2011, Ryskov et al. 2017). We captured and identified 17 individuals in 10 SZs. All individuals were found to be females, determined by visual inspection of the genitals.

*Darevskia rostombekowi* has a relatively small range, consisting of several different isolated areas within northern Armenia, north-western Azerbaijan, the territory of Nagorno-Karabakh and a small alpine relict isolated area from the main range on the south-eastern coast of Lake Sevan (Macculloch et al. 1997, Arakelyan et al. 2011). *Darevskia rostombekowi* has at least two SZs with both parental species *D. portschinskii* and *D. r. raddei* in the two north-western provinces of Tavush and Lori in Armenia. The number of SZs, where there was co-existence with other parthenogenetic forms of *D. armeniaca*, *D. dahli* and *D. unisexualis*, are four, three and two, respectively. In general, SZs with other species of the genus *Darevskia* located in the forest zone, mountain meadows, mountain steppes and anthropogenic transformed habitats, were identified within the range of the species.

### Darevskia unisexualis

(Darevsky, 1966)

144992FF-575E-5926-976D-771AD9A4A0BD

#### Materials

- **Type status:** Other material. **Occurrence:** catalogNumber: REPAMPHRU2018390; recordedBy: Osipov F.A.; individualCount: 1; sex: fmale; lifeStage: adult; occurrenceID: urn:IEERASBIOINF:REPAMPHRU2018390; **Taxon:** scientificName: Darevskia unisexualis; kingdom: Animalia ; phylum: Chordata ; class: Reptilia ; order: Squamata ; family: Lacertidae ; genus: Darevskia ; scientificNameAuthorship: Darevsky, 1966; **Location:** country: Armenia; stateProvince: Kotayk Province; locality: Punick; decimalLatitude: 40.609193; decimalLongitude: 44.60197; geodeticDatum: WGS1984; georeferenceProtocol: GPS; **Identification:** identifiedBy: Arakelyan M.S.; **Event:** samplingProtocol: Captured by noose; eventDate: 2018-7-8; **Record Level:** language: en; rights: https://creativecommons.org/publicdomain/zero/1.0/; rightsHolder: Petrosyan V.G.; accessRights: http://vertnet.org/resources/norms.html; institutionCode: IEERASBIOINF; collectionCode: REPAMPHRU; basisOfRecord: HumanObservation
- **Type status:** Other material. **Occurrence:** catalogNumber: REPAMPHRU2018402; recordedBy: Osipov F.A.; individualCount: 2; sex: fmale; lifeStage: adult; occurrenceID: urn:IEERASBIOINF:REPAMPHRU2018402; **Taxon:** scientificName: Darevskia unisexualis; kingdom: Animalia ; phylum: Chordata ; class: Reptilia ; order: Squamata ; family: Lacertidae ; genus: Darevskia ; scientificNameAuthorship: Darevsky, 1966; **Location:** country: Armenia; stateProvince: Kotayk Province; locality: Hrazdan city; decimalLatitude: 40.506393; decimalLongitude: 44.748776; geodeticDatum: WGS1984; georeferenceProtocol: GPS; **Identification:** identifiedBy: Arakelyan M.S.; **Event:** samplingProtocol: Captured by noose; eventDate: 2018-7-9; **Record Level:** language: en; rights: https://creativecommons.org/publicdomain/zero/1.0/; rightsHolder: Petrosyan V.G.; accessRights: http://vertnet.org/resources/norms.html; institutionCode: IEERASBIOINF; collectionCode: REPAMPHRU; basisOfRecord: HumanObservation
- **Type status:** Other material. **Occurrence:** catalogNumber: REPAMPHRU2018405; recordedBy: Osipov F.A.; individualCount: 1; sex: fmale; lifeStage: adult; occurrenceID: urn:IEERASBIOINF:REPAMPHRU2018405; **Taxon:** scientificName: Darevskia unisexualis; kingdom: Animalia ; phylum: Chordata ; class: Reptilia ; order: Squamata ; family: Lacertidae ; genus: Darevskia ; scientificNameAuthorship: Darevsky, 1966; **Location:** country: Armenia; stateProvince: Gegharkunik Province; locality: Tsovak; decimalLatitude: 40.185681; decimalLongitude: 45.623972; geodeticDatum: WGS1984; georeferenceProtocol: GPS; **Identification:** identifiedBy: Arakelyan M.S.; **Event:** samplingProtocol: Captured by noose; eventDate: 2018-7-10; **Record Level:** language: en; rights: https://creativecommons.org/publicdomain/zero/1.0/; rightsHolder: Petrosyan V.G.; accessRights: http://vertnet.org/resources/norms.html; institutionCode: IEERASBIOINF; collectionCode: REPAMPHRU; basisOfRecord: HumanObservation
- **Type status:** Other material. **Occurrence:** catalogNumber: REPAMPHRU2018410; recordedBy: Osipov F.A.; individualCount: 1; sex: fmale; lifeStage: adult; occurrenceID: urn:IEERASBIOINF:REPAMPHRU2018410; **Taxon:** scientificName: Darevskia unisexualis; kingdom: Animalia ; phylum: Chordata ; class: Reptilia ; order: Squamata ; family: Lacertidae ; genus: Darevskia ; scientificNameAuthorship: Darevsky, 1966; **Location:** country: Armenia; stateProvince: Aragatsotn Province; locality: Kuchak; decimalLatitude: 40.528691; decimalLongitude: 44.388427; geodeticDatum: WGS1984; georeferenceProtocol: GPS; **Identification:** identifiedBy: Arakelyan M.S.; **Event:** samplingProtocol: Captured by noose; eventDate: 2018-7-11; **Record Level:** language: en; rights: https://creativecommons.org/publicdomain/zero/1.0/; rightsHolder: Petrosyan V.G.; accessRights: http://vertnet.org/resources/norms.html; institutionCode: IEERASBIOINF; collectionCode: REPAMPHRU; basisOfRecord: HumanObservation
- **Type status:** Other material. **Occurrence:** catalogNumber: REPAMPHRU2019416; recordedBy: Osipov F.A.; individualCount: 1; sex: fmale; lifeStage: adult; occurrenceID: urn:IEERASBIOINF:REPAMPHRU2019416; **Taxon:** scientificName: Darevskia unisexualis; kingdom: Animalia ; phylum: Chordata ; class: Reptilia ; order: Squamata ; family: Lacertidae ; genus: Darevskia ; scientificNameAuthorship: Darevsky, 1966; **Location:** country: Armenia; stateProvince: Aragatsotn Province; locality: Tsilkar; decimalLatitude: 40.736893; decimalLongitude: 44.197427; geodeticDatum: WGS1984; georeferenceProtocol: GPS; **Identification:** identifiedBy: Arakelyan M.S.; **Event:** samplingProtocol: Captured by noose; eventDate: 2019-7-16; **Record Level:** language: en; rights: https://creativecommons.org/publicdomain/zero/1.0/; rightsHolder: Petrosyan V.G.; accessRights: http://vertnet.org/resources/norms.html; institutionCode: IEERASBIOINF; collectionCode: REPAMPHRU; basisOfRecord: HumanObservation
- **Type status:** Other material. **Occurrence:** catalogNumber: REPAMPHRU2019429; recordedBy: Osipov F.A.; individualCount: 1; sex: fmale; lifeStage: adult; occurrenceID: urn:IEERASBIOINF:REPAMPHRU2019429; **Taxon:** scientificName: Darevskia unisexualis; kingdom: Animalia ; phylum: Chordata ; class: Reptilia ; order: Squamata ; family: Lacertidae ; genus: Darevskia ; scientificNameAuthorship: Darevsky, 1966; **Location:** country: Armenia; stateProvince: Gegharkunik Province; locality: Close to Noratus; decimalLatitude: 40.39722; decimalLongitude: 45.144424; geodeticDatum: WGS1984; georeferenceProtocol: GPS; **Identification:** identifiedBy: Arakelyan M.S.; **Event:** samplingProtocol: Captured by noose; eventDate: 2019-7-19; **Record Level:** language: en; rights: https://creativecommons.org/publicdomain/zero/1.0/; rightsHolder: Petrosyan V.G.; accessRights: http://vertnet.org/resources/norms.html; institutionCode: IEERASBIOINF; collectionCode: REPAMPHRU; basisOfRecord: HumanObservation
- **Type status:** Other material. **Occurrence:** catalogNumber: REPAMPHRU2019441; recordedBy: Osipov F.A.; individualCount: 4; sex: fmale; lifeStage: adult; occurrenceID: urn:IEERASBIOINF:REPAMPHRU2019441; **Taxon:** scientificName: Darevskia unisexualis; kingdom: Animalia ; phylum: Chordata ; class: Reptilia ; order: Squamata ; family: Lacertidae ; genus: Darevskia ; scientificNameAuthorship: Darevsky, 1966; **Location:** country: Armenia; stateProvince: Shirak Province; locality: Keti; decimalLatitude: 40.864013; decimalLongitude: 43.841847; geodeticDatum: WGS1984; georeferenceProtocol: GPS; **Identification:** identifiedBy: Arakelyan M.S.; **Event:** samplingProtocol: Captured by noose; eventDate: 2019-7-25; **Record Level:** language: en; rights: https://creativecommons.org/publicdomain/zero/1.0/; rightsHolder: Petrosyan V.G.; accessRights: http://vertnet.org/resources/norms.html; institutionCode: IEERASBIOINF; collectionCode: REPAMPHRU; basisOfRecord: HumanObservation
- **Type status:** Other material. **Occurrence:** catalogNumber: REPAMPHRU2019459; recordedBy: Osipov F.A.; individualCount: 3; sex: fmale; lifeStage: adult; occurrenceID: urn:IEERASBIOINF:REPAMPHRU2019459; **Taxon:** scientificName: Darevskia unisexualis; kingdom: Animalia ; phylum: Chordata ; class: Reptilia ; order: Squamata ; family: Lacertidae ; genus: Darevskia ; scientificNameAuthorship: Darevsky, 1966; **Location:** country: Armenia; stateProvince: Kotayk Province; locality: Tsakhkadzor; decimalLatitude: 40.53515; decimalLongitude: 44.6972; geodeticDatum: WGS1984; georeferenceProtocol: GPS; **Identification:** identifiedBy: Arakelyan M.S.; **Event:** samplingProtocol: Captured by noose; eventDate: 2018-7-9; **Record Level:** language: en; rights: https://creativecommons.org/publicdomain/zero/1.0/; rightsHolder: Petrosyan V.G.; accessRights: http://vertnet.org/resources/norms.html; institutionCode: IEERASBIOINF; collectionCode: REPAMPHRU; basisOfRecord: HumanObservation
- **Type status:** Other material. **Occurrence:** catalogNumber: REPAMPHRU2019463; recordedBy: Osipov F.A.; individualCount: 2; sex: fmale; lifeStage: adult; occurrenceID: urn:IEERASBIOINF:REPAMPHRU2019463; **Taxon:** scientificName: Darevskia unisexualis; kingdom: Animalia ; phylum: Chordata ; class: Reptilia ; order: Squamata ; family: Lacertidae ; genus: Darevskia ; scientificNameAuthorship: Darevsky, 1966; **Location:** country: Armenia; stateProvince: Kotayk Province; locality: Artavazd; decimalLatitude: 40.620316666667; decimalLongitude: 44.56305; geodeticDatum: WGS1984; georeferenceProtocol: GPS; **Identification:** identifiedBy: Arakelyan M.S.; **Event:** samplingProtocol: Captured by noose; eventDate: 2018-7-14; **Record Level:** language: en; rights: https://creativecommons.org/publicdomain/zero/1.0/; rightsHolder: Petrosyan V.G.; accessRights: http://vertnet.org/resources/norms.html; institutionCode: IEERASBIOINF; collectionCode: REPAMPHRU; basisOfRecord: HumanObservation
- **Type status:** Other material. **Occurrence:** catalogNumber: REPAMPHRU2019477; recordedBy: Osipov F.A.; individualCount: 1; sex: fmale; lifeStage: adult; occurrenceID: urn:IEERASBIOINF:REPAMPHRU2019477; **Taxon:** scientificName: Darevskia unisexualis; kingdom: Animalia ; phylum: Chordata ; class: Reptilia ; order: Squamata ; family: Lacertidae ; genus: Darevskia ; scientificNameAuthorship: Darevsky, 1966; **Location:** country: Armenia; stateProvince: Gegharkunik Province; locality: Lchap, Sevan Lake; decimalLatitude: 40.4673; decimalLongitude: 45.0621; geodeticDatum: WGS1984; georeferenceProtocol: GPS; **Identification:** identifiedBy: Arakelyan M.S.; **Event:** samplingProtocol: Captured by noose; eventDate: 2019-6-20; **Record Level:** language: en; rights: https://creativecommons.org/publicdomain/zero/1.0/; rightsHolder: Petrosyan V.G.; accessRights: http://vertnet.org/resources/norms.html; institutionCode: IEERASBIOINF; collectionCode: REPAMPHRU; basisOfRecord: HumanObservation
- **Type status:** Other material. **Occurrence:** catalogNumber: REPAMPHRU2019479; recordedBy: Osipov F.A.; individualCount: 1; sex: fmale; lifeStage: adult; occurrenceID: urn:IEERASBIOINF:REPAMPHRU2019479; **Taxon:** scientificName: Darevskia unisexualis; kingdom: Animalia ; phylum: Chordata ; class: Reptilia ; order: Squamata ; family: Lacertidae ; genus: Darevskia ; scientificNameAuthorship: Darevsky, 1966; **Location:** country: Armenia; stateProvince: Lori Province; locality: Spitak; decimalLatitude: 40.8249; decimalLongitude: 44.2775; geodeticDatum: WGS1984; georeferenceProtocol: GPS; **Identification:** identifiedBy: Arakelyan M.S.; **Event:** samplingProtocol: Captured by noose; eventDate: 2019-6-20; **Record Level:** language: en; rights: https://creativecommons.org/publicdomain/zero/1.0/; rightsHolder: Petrosyan V.G.; accessRights: http://vertnet.org/resources/norms.html; institutionCode: IEERASBIOINF; collectionCode: REPAMPHRU; basisOfRecord: HumanObservation

#### Notes

**Figure 6.**
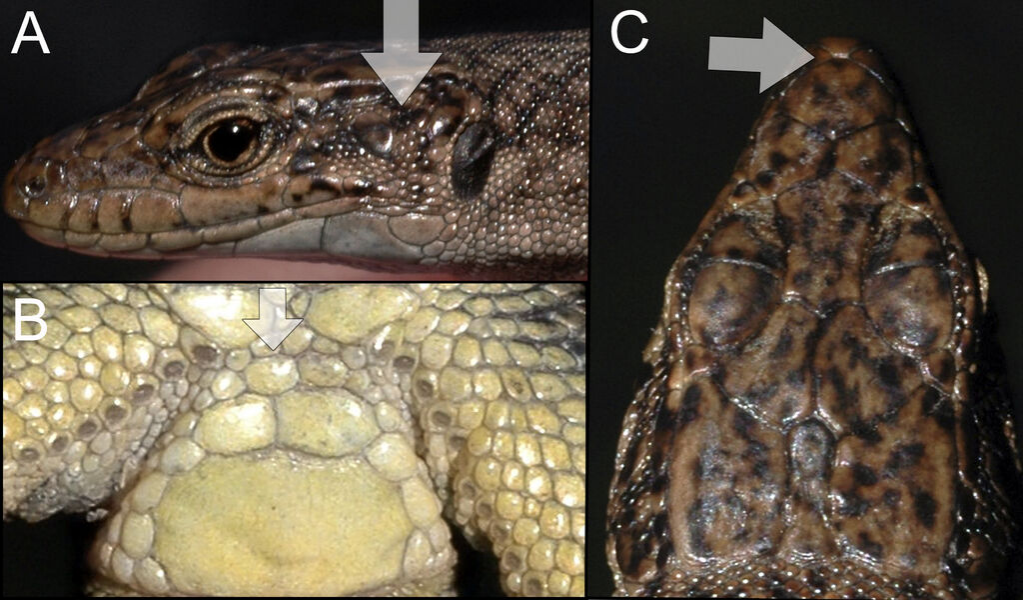
Distinguishing characters of *D. unisexualis*. **A.** The central temporal shield is large, often divided into two; from the first upper temporal shield in the narrowest place, it is separated by 1-3 and from the tympanum - by 2-4 transverse rows of enlarged shields (indicated by arrow); **B.** The anal shield is large, in front of it four small and approximately equal in size pre-anal shields are located symmetrically, the middle of which can be slightly increased. (indicated by arrow); **C.** Only in this species - the maxillary shield is in contact with the fronto-nasal (indicated by arrow). Photos by I. Kropachev.

The parthenogenetic lizard of *D. unisexualis* is formed as a result of interspecific hybridisation between bisexual *D. valentini* ("paternal") and *D. r. nairensis* ("maternal") species (Darevsky 1966, Darevsky 1967, Uzzell and Darevsky 1975, Moritz et al. 1992, Murphy et al. 2000). Species identification in studed SZs was carried out using allozyme loci, mt-DNA, multilocus DNA fingerprinting, mini- and micro-satellite markers and morphological traits (Darevsky 1967, Uzzell and Darevsky 1975, Murphy et al. 2000, Ryskov et al. 2000, Tokarskaya et al. 2001, Ryskov et al. 2003, Tokarskaya et al. 2003, Korchagin and Tokarskaya 2010, Omel'chenko et al. 2009a, Omel'chenko et al. 2009b, Arakelyan et al. 2011). In the field, *D. unisexualis* was identified using species identification guides according to Darevsky (Darevsky 1967), (Fig. 6), since it has previously been shown that the identification using specific features did not cause confusion between the species (Darevsky 1967, Uzzell and Darevsky 1975, Tokarskaya et al. 2001, Tokarskaya et al. 2003, Omel'chenko et al. 2009a, Omel'chenko et al. 2009b, Arakelyan et al. 2011). We captured and identified 18 individuals in 11 SZs. All individuals were found to be females, determined by visual inspection of the genitals.

The *D. unisexualis* range covers the territory of northern and central highland Armenia and the adjacent regions of eastern Turkey. In Armenia, the species is found in isolated populations in the five Provinces of Aragatsotn, Gegharkunik, Kotayk, Lori and Shirak. In these Provinces, nine SZs were identified, including four SZs in which *D. unisexualis* co-exists with the "maternal" species *D. r. nairensis* and in two SZs with the "paternal" species *D. valentini*. The number of SZs, where there is co-existence with other parthenogenetic species *D. armeniaca*, *D. dahli* and *D. rostombekowi*, are five, two and two, respectively. Hybrid individuals of *D. valentini* x *D. unisexualis* were found in two zones (Artavazd, Kuchak), which were previously noted in literature (Danielyan et al. 2008,Spangenberg et al. 2017, Carretero et al. 2018). In the SZ Artavazd, autotriploid males and sterile intersexual individuals were found from 1984-1988 (Darevsky et al. 1989). The origin of these hybrids can be explained by interclonal mating between parthenogenetic females and rare, conspecific diploid males. Identified SZs are located at rock outcrops, piles of stones and rocky slopes in the mountain-steppe zone.

### Darevskia raddei raddei

(Boettger, 1892)

46E21B73-1E2E-50E2-A466-C5047251FAE2

#### Materials

- **Type status:** Other material. **Occurrence:** catalogNumber: REPAMPHRU2019436; recordedBy: Osipov F.A.; individualCount: 1; sex: male; lifeStage: adult; occurrenceID: urn:IEERASBIOINF:REPAMPHRU2019436; **Taxon:** scientificName: Darevskia raddei raddei; kingdom: Animalia; phylum: Chordata; class: Reptilia; order: Squamata; family: Lacertidae; genus: Darevskia; scientificNameAuthorship: Boettger, 1892; **Location:** country: Armenia; stateProvince: Tavush Province; locality: Forest area near Acharkut; decimalLatitude: 41.026279; decimalLongitude: 45.052574; geodeticDatum: WGS1984; georeferenceProtocol: GPS; **Identification:** identifiedBy: Arakelyan M.S.; **Event:** samplingProtocol: Captured by noose; eventDate: 07/23/2019; **Record Level:** language: en; rights: https://creativecommons.org/publicdomain/zero/1.0/; rightsHolder: Petrosyan V.G.; accessRights: http://vertnet.org/resources/norms.html; institutionCode: IEERASBIOINF; collectionCode: REPAMPHRU; basisOfRecord: HumanObservation
- **Type status:** Other material. **Occurrence:** catalogNumber: REPAMPHRU2019452; recordedBy: Osipov F.A.; individualCount: 3; sex: fmale; lifeStage: adult; occurrenceID: urn:IEERASBIOINF:REPAMPHRU2019452; **Taxon:** scientificName: Darevskia raddei raddei; kingdom: Animalia; phylum: Chordata; class: Reptilia; order: Squamata; family: Lacertidae; genus: Darevskia; scientificNameAuthorship: Boettger, 1892; **Location:** country: Nagorno-Karabakh; stateProvince: Shahumyan Province; locality: Zuar; decimalLatitude: 40.047933333333; decimalLongitude: 46.2367; geodeticDatum: WGS1984; georeferenceProtocol: GPS; **Identification:** identifiedBy: Arakelyan M.S.; **Event:** samplingProtocol: Captured by noose; eventDate: 07/10/2018; **Record Level:** language: en; rights: https://creativecommons.org/publicdomain/zero/1.0/; rightsHolder: Petrosyan V.G.; accessRights: http://vertnet.org/resources/norms.html; institutionCode: IEERASBIOINF; collectionCode: REPAMPHRU; basisOfRecord: HumanObservation
- **Type status:** Other material. **Occurrence:** catalogNumber: REPAMPHRU2019454; recordedBy: Osipov F.A.; individualCount: 4; sex: fmale; lifeStage: adult; occurrenceID: urn:IEERASBIOINF:REPAMPHRU2019454; **Taxon:** scientificName: Darevskia raddei raddei; kingdom: Animalia; phylum: Chordata; class: Reptilia; order: Squamata; family: Lacertidae; genus: Darevskia; scientificNameAuthorship: Boettger, 1892; **Location:** country: Armenia; stateProvince: Tavush Province; locality: Gosh; decimalLatitude: 40.73916667; decimalLongitude: 45.02076667; geodeticDatum: WGS1984; georeferenceProtocol: GPS; **Identification:** identifiedBy: Arakelyan M.S.; **Event:** samplingProtocol: Captured by noose; eventDate: 07/21/2018; **Record Level:** language: en; rights: https://creativecommons.org/publicdomain/zero/1.0/; rightsHolder: Petrosyan V.G.; accessRights: http://vertnet.org/resources/norms.html; institutionCode: IEERASBIOINF; collectionCode: REPAMPHRU; basisOfRecord: HumanObservation
- **Type status:** Other material. **Occurrence:** catalogNumber: REPAMPHRU2019456; recordedBy: Osipov F.A.; individualCount: 1; sex: male; lifeStage: adult; occurrenceID: urn:IEERASBIOINF:REPAMPHRU2019456; **Taxon:** scientificName: Darevskia raddei raddei; kingdom: Animalia; phylum: Chordata; class: Reptilia; order: Squamata; family: Lacertidae; genus: Darevskia; scientificNameAuthorship: Boettger, 1892; **Location:** country: Armenia; stateProvince: Tavush Province; locality: Goshavank monastery; decimalLatitude: 40.729851666667; decimalLongitude: 44.99711; geodeticDatum: WGS1984; georeferenceProtocol: GPS; **Identification:** identifiedBy: Arakelyan M.S.; **Event:** samplingProtocol: Captured by noose; eventDate: 07/21/2018; **Record Level:** language: en; rights: https://creativecommons.org/publicdomain/zero/1.0/; rightsHolder: Petrosyan V.G.; accessRights: http://vertnet.org/resources/norms.html; institutionCode: IEERASBIOINF; collectionCode: REPAMPHRU; basisOfRecord: HumanObservation
- **Type status:** Other material. **Occurrence:** catalogNumber: REPAMPHRU2019460; recordedBy: Osipov F.A.; individualCount: 2; sex: fmale; lifeStage: adult; occurrenceID: urn:IEERASBIOINF:REPAMPHRU2019460; **Taxon:** scientificName: Darevskia raddei raddei; kingdom: Animalia; phylum: Chordata; class: Reptilia; order: Squamata; family: Lacertidae; genus: Darevskia; scientificNameAuthorship: Boettger, 1892; **Location:** country: Nagorno-Karabakh; stateProvince: Shahumyan Province; locality: Road to Karvachar; decimalLatitude: 40.096783333333; decimalLongitude: 46.0338; geodeticDatum: WGS1984; georeferenceProtocol: GPS; **Identification:** identifiedBy: Arakelyan M.S.; **Event:** samplingProtocol: Captured by noose; eventDate: 07/10/2018; **Record Level:** language: en; rights: https://creativecommons.org/publicdomain/zero/1.0/; rightsHolder: Petrosyan V.G.; accessRights: http://vertnet.org/resources/norms.html; institutionCode: IEERASBIOINF; collectionCode: REPAMPHRU; basisOfRecord: HumanObservation

#### Notes

**Figure 7.**
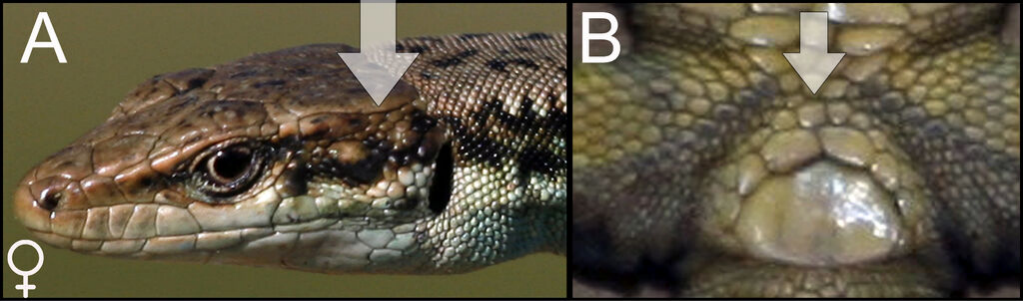
Distinguishing characters of *D. r. raddei* (female individual on the photographs). **A.** Between the average size central temporal and small tympanum shield in the narrowest place, there are 2-5 small shields (indicated by arrow); **B.** The anal shield is large, in front of it, there are two more or less enlarged pre-anals located symmetrically, between which a small third one is often wedged (indicated by arrow). Photos by I. Kropachev.

The bisexual species, *D. raddei*, is considered as a complex (*Darevskia raddei* sensu lato) containing four forms (subspecies) “raddei”, “nairensis”, “vanensis” and “chaldoranensis” (Grechko et al. 2007, Rastegar-Pouyani et al. 2012, Omelchenko et al. 2016, Freitas et al. 2016). However, the subspecies “raddei” (*D. r. raddei*) is the only form widely distributed in Armenia, Nagorno-Karabakh, Azerbaijan and the northern province of Ardabil of Iran (Freitas et al. 2016). Identification of *D. r. raddei* individuals in different SZs was undertaken by authors using allozyme markers, mt-DNA, multilocus DNA fingerprinting, mini- and micro-satellite markers and morphological traits (Bobyn et al. 1996, Fu et al. 2000a, Grechko et al. 2007, Korchagin et al. 2007, Korchagin and Tokarskaya 2010, Omelchenko et al. 2016, Freitas et al. 2016). In the field, *D. r. raddei* was identified using identification guides according to Darevsky (Darevsky 1967) (Fig. 7). During the field survey, we captured and identified 11 individuals, including nine adult females and two males.

The bisexual form of *D. r. raddei* is widespread in southern Armenia, north-eastern Turkey, north-western regions of Nagorno-Karabakh, south and south-western Azerbaijan and in adjacent regions of north-western Iran (Darevsky 1967). During the field survey, five SZs were identified with other parthenogenetic and bisexual species in isolated populations in north-eastern Armenia and Nagorno-Karabakh. Three SZs exist in north-eastern Armenia in the Tavush Province and two zones in Nagorno-Karabakh (Shahumyan Province). This species in the two SZs co-exists with the bisexual species *D. portschinskii* and in two SZs with the "daughter" species, *D. rostombekowi*. The identified SZs are located in rocky habitats in forest and mountain steppes, including the stone walls of buildings (e.g. on the walls of churches) and stone ruins.

### Darevskia raddei nairensis

(Darevsky, 1967)

7A689ECC-4E24-59C8-BBE0-F2F9237644F5

#### Materials

- **Type status:** Other material. **Occurrence:** catalogNumber: REPAMPHRU2018387; recordedBy: Osipov F.A.; individualCount: 3; sex: fmale; lifeStage: adult; occurrenceID: urn:IEERASBIOINF:REPAMPHRU2018387; **Taxon:** scientificName: Darevskia raddei nairensis; kingdom: Animalia; phylum: Chordata; class: Reptilia; order: Squamata; family: Lacertidae; genus: Darevskia; scientificNameAuthorship: Darevsky, 1967; **Location:** country: Armenia; stateProvince: Gegharkunik Province; locality: Lchashen, Sevan lake; decimalLatitude: 40.510698; decimalLongitude: 44.935422; geodeticDatum: WGS1984; georeferenceProtocol: GPS; **Identification:** identifiedBy: Arakelyan M.S.; **Event:** samplingProtocol: Captured by noose; eventDate: 07/08/2018; **Record Level:** language: en; rights: https://creativecommons.org/publicdomain/zero/1.0/; rightsHolder: Petrosyan V.G.; accessRights: http://vertnet.org/resources/norms.html; institutionCode: IEERASBIOINF; collectionCode: REPAMPHRU; basisOfRecord: HumanObservation
- **Type status:** Other material. **Occurrence:** catalogNumber: REPAMPHRU2018391; recordedBy: Osipov F.A.; individualCount: 1; sex: fmale; lifeStage: adult; occurrenceID: urn:IEERASBIOINF:REPAMPHRU2018391; **Taxon:** scientificName: Darevskia raddei nairensis; kingdom: Animalia; phylum: Chordata; class: Reptilia; order: Squamata; family: Lacertidae; genus: Darevskia; scientificNameAuthorship: Darevsky, 1967; **Location:** country: Armenia; stateProvince: Kotayk Province; locality: Punick; decimalLatitude: 40.609193; decimalLongitude: 44.60197; geodeticDatum: WGS1984; georeferenceProtocol: GPS; **Identification:** identifiedBy: Arakelyan M.S.; **Event:** samplingProtocol: Captured by noose; eventDate: 07/08/2018; **Record Level:** language: en; rights: https://creativecommons.org/publicdomain/zero/1.0/; rightsHolder: Petrosyan V.G.; accessRights: http://vertnet.org/resources/norms.html; institutionCode: IEERASBIOINF; collectionCode: REPAMPHRU; basisOfRecord: HumanObservation
- **Type status:** Other material. **Occurrence:** catalogNumber: REPAMPHRU2018403; recordedBy: Osipov F.A.; individualCount: 2; sex: male; lifeStage: adult; occurrenceID: urn:IEERASBIOINF:REPAMPHRU2018403; **Taxon:** scientificName: Darevskia raddei nairensis; kingdom: Animalia; phylum: Chordata; class: Reptilia; order: Squamata; family: Lacertidae; genus: Darevskia; scientificNameAuthorship: Darevsky, 1967; **Location:** country: Armenia; stateProvince: Kotayk Province; locality: Hrazdan city; decimalLatitude: 40.506393; decimalLongitude: 44.748776; geodeticDatum: WGS1984; georeferenceProtocol: GPS; **Identification:** identifiedBy: Arakelyan M.S.; **Event:** samplingProtocol: Captured by noose; eventDate: 07/09/2018; **Record Level:** language: en; rights: https://creativecommons.org/publicdomain/zero/1.0/; rightsHolder: Petrosyan V.G.; accessRights: http://vertnet.org/resources/norms.html; institutionCode: IEERASBIOINF; collectionCode: REPAMPHRU; basisOfRecord: HumanObservation
- **Type status:** Other material. **Occurrence:** catalogNumber: REPAMPHRU2019428; recordedBy: Osipov F.A.; individualCount: 1; sex: male; lifeStage: adult; occurrenceID: urn:IEERASBIOINF:REPAMPHRU2019428; **Taxon:** scientificName: Darevskia raddei nairensis; kingdom: Animalia; phylum: Chordata; class: Reptilia; order: Squamata; family: Lacertidae; genus: Darevskia; scientificNameAuthorship: Darevsky, 1967; **Location:** country: Armenia; stateProvince: Gegharkunik Province; locality: Close to Noratus; decimalLatitude: 40.39722; decimalLongitude: 45.144424; geodeticDatum: WGS1984; georeferenceProtocol: GPS; **Identification:** identifiedBy: Arakelyan M.S.; **Event:** samplingProtocol: Captured by noose; eventDate: 07/19/2019; **Record Level:** language: en; rights: https://creativecommons.org/publicdomain/zero/1.0/; rightsHolder: Petrosyan V.G.; accessRights: http://vertnet.org/resources/norms.html; institutionCode: IEERASBIOINF; collectionCode: REPAMPHRU; basisOfRecord: HumanObservation
- **Type status:** Other material. **Occurrence:** catalogNumber: REPAMPHRU2019442; recordedBy: Osipov F.A.; individualCount: 5; sex: fmale; lifeStage: adult; occurrenceID: urn:IEERASBIOINF:REPAMPHRU2019442; **Taxon:** scientificName: Darevskia raddei nairensis; kingdom: Animalia; phylum: Chordata; class: Reptilia; order: Squamata; family: Lacertidae; genus: Darevskia; scientificNameAuthorship: Darevsky, 1967; **Location:** country: Armenia; stateProvince: Shirak Province; locality: Keti; decimalLatitude: 40.864013; decimalLongitude: 43.841847; geodeticDatum: WGS1984; georeferenceProtocol: GPS; **Identification:** identifiedBy: Arakelyan M.S.; **Event:** samplingProtocol: Captured by noose; eventDate: 07/25/2019; **Record Level:** language: en; rights: https://creativecommons.org/publicdomain/zero/1.0/; rightsHolder: Petrosyan V.G.; accessRights: http://vertnet.org/resources/norms.html; institutionCode: IEERASBIOINF; collectionCode: REPAMPHRU; basisOfRecord: HumanObservation
- **Type status:** Other material. **Occurrence:** catalogNumber: REPAMPHRU2019478; recordedBy: Osipov F.A.; individualCount: 1; sex: fmale; lifeStage: adult; occurrenceID: urn:IEERASBIOINF:REPAMPHRU2019478; **Taxon:** scientificName: Darevskia raddei nairensis; kingdom: Animalia; phylum: Chordata; class: Reptilia; order: Squamata; family: Lacertidae; genus: Darevskia; scientificNameAuthorship: Darevsky, 1967; **Location:** country: Armenia; stateProvince: Gegharkunik Province; locality: Lchap, Sevan Lake; decimalLatitude: 40.4673; decimalLongitude: 45.0621; geodeticDatum: WGS1984; georeferenceProtocol: GPS; **Identification:** identifiedBy: Arakelyan M.S.; **Event:** samplingProtocol: Captured by noose; eventDate: 06/20/2019; **Record Level:** language: en; rights: https://creativecommons.org/publicdomain/zero/1.0/; rightsHolder: Petrosyan V.G.; accessRights: http://vertnet.org/resources/norms.html; institutionCode: IEERASBIOINF; collectionCode: REPAMPHRU; basisOfRecord: HumanObservation

#### Notes

**Figure 8.**
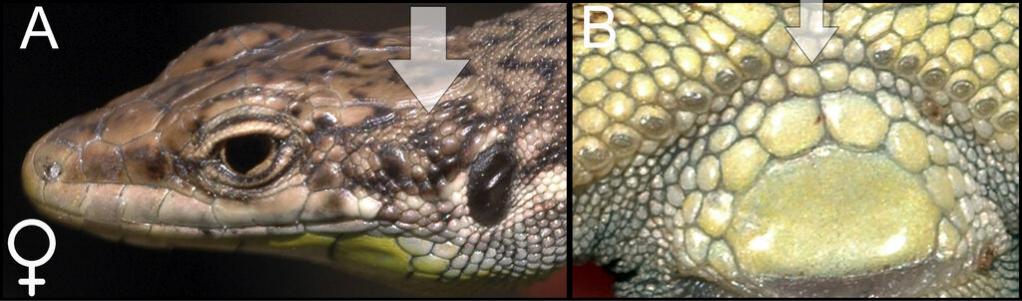
Distinguishing characters of *D. r. nairensis* (female individual on the photographs). **A.** Central temporal shield is small. Between the central temporal and rather large tympanic shields on the sides of the head, there are two or three enlarged shields (indicated by arrow); **B.** In front of a large, elongated transverse anal shield, there are two large pre-anal ones (indicated by arrow). Photos by I. Kropachev.

Although early studies found very low genetic differences between *D. r. raddei* and *D. r. nairensis* (*Bobyn et al. 1996, Fu et al. 2000a*), later studies ([Bibr B5882290][Bibr B5882924], Omelchenko et al. 2016), however, considered them as two subspecies. *Darevskia r. raddei* and *D. r. nairensis* were considered as different subspecies, since they are reproductively isolated in terms of distinctive periods of mating (Danielyan 1989). In the field, *D. r. nairensis* was identified using identification guides according to Darevsky (Darevsky 1967), (Fig. 8). During the field survey, we captured and identified 13 individuals, including ten adult females and three males.

The range of *D. r. nairensis* covers central, south-western Armenia, the western shore of Lake Sevan and is restricted to the north-eastern part of Armenia. This species also inhabits neighbouring regions of north-eastern Turkey and southern Georgia. In Armenia, there are several isolated populations in the north Provinces (Tumanyan and Lori) and in the south-central part (Karmrashen and Vayots Dzor Provinces). During the field survey, six SZs were identified in the three Provinces of Gegharkunik, Kotayk and Shirak. This species has a relatively large number of SZs - four with a "daughter" species *D. unisexualis* and, with other parthenogenetic species, *D. armeniaca* and *D. dahli*, there are two and one SZs, respectively. Our data show that *D. r. nairensis* has only one SZ with a bisexual species *D. valentini*. The SZs are mainly located in the rocky habitats of the mountain steppe.

### Darevskia valentini

(Boettger, 1892)

24FDA931-117A-5A9B-B821-28FF8E7403A3

#### Materials

- **Type status:** Other material. **Occurrence:** catalogNumber: REPAMPHRU2018388; recordedBy: Osipov F.A.; individualCount: 2; sex: fmale; lifeStage: adult; occurrenceID: urn:IEERASBIOINF:REPAMPHRU2018388; **Taxon:** scientificName: Darevskia valentini; kingdom: Animalia ; phylum: Chordata ; class: Reptilia ; order: Squamata ; family: Lacertidae ; genus: Darevskia ; scientificNameAuthorship: Boettger, 1892; **Location:** country: Armenia; stateProvince: Gegharkunik Province; locality: Lchashen, Sevan lake; decimalLatitude: 40.510698; decimalLongitude: 44.935422; geodeticDatum: WGS1984; georeferenceProtocol: GPS; **Identification:** identifiedBy: Arakelyan M.S.; **Event:** samplingProtocol: Captured by noose; eventDate: 2018-7-8; **Record Level:** language: en; rights: https://creativecommons.org/publicdomain/zero/1.0/; rightsHolder: Petrosyan V.G.; accessRights: http://vertnet.org/resources/norms.html; institutionCode: IEERASBIOINF; collectionCode: REPAMPHRU; basisOfRecord: HumanObservation
- **Type status:** Other material. **Occurrence:** catalogNumber: REPAMPHRU2018406; recordedBy: Osipov F.A.; individualCount: 3; sex: fmale; lifeStage: adult; occurrenceID: urn:IEERASBIOINF:REPAMPHRU2018406; **Taxon:** scientificName: Darevskia valentini; kingdom: Animalia ; phylum: Chordata ; class: Reptilia ; order: Squamata ; family: Lacertidae ; genus: Darevskia ; scientificNameAuthorship: Boettger, 1892; **Location:** country: Armenia; stateProvince: Gegharkunik Province; locality: Karabakh–Sotk road; decimalLatitude: 40.223085; decimalLongitude: 46.00103; geodeticDatum: WGS1984; georeferenceProtocol: GPS; **Identification:** identifiedBy: Arakelyan M.S.; **Event:** samplingProtocol: Captured by noose; eventDate: 2018-7-10; **Record Level:** language: en; rights: https://creativecommons.org/publicdomain/zero/1.0/; rightsHolder: Petrosyan V.G.; accessRights: http://vertnet.org/resources/norms.html; institutionCode: IEERASBIOINF; collectionCode: REPAMPHRU; basisOfRecord: HumanObservation
- **Type status:** Other material. **Occurrence:** catalogNumber: REPAMPHRU2018409; recordedBy: Osipov F.A.; individualCount: 1; sex: fmale; lifeStage: adult; occurrenceID: urn:IEERASBIOINF:REPAMPHRU2018409; **Taxon:** scientificName: Darevskia valentini; kingdom: Animalia ; phylum: Chordata ; class: Reptilia ; order: Squamata ; family: Lacertidae ; genus: Darevskia ; scientificNameAuthorship: Boettger, 1892; **Location:** country: Armenia; stateProvince: Aragatsotn Province; locality: Kuchak; decimalLatitude: 40.528691; decimalLongitude: 44.388427; geodeticDatum: WGS1984; georeferenceProtocol: GPS; **Identification:** identifiedBy: Arakelyan M.S.; **Event:** samplingProtocol: Captured by noose; eventDate: 2018-7-11; **Record Level:** language: en; rights: https://creativecommons.org/publicdomain/zero/1.0/; rightsHolder: Petrosyan V.G.; accessRights: http://vertnet.org/resources/norms.html; institutionCode: IEERASBIOINF; collectionCode: REPAMPHRU; basisOfRecord: HumanObservation
- **Type status:** Other material. **Occurrence:** catalogNumber: REPAMPHRU2018412; recordedBy: Osipov F.A.; individualCount: 2; sex: male; lifeStage: adult; occurrenceID: urn:IEERASBIOINF:REPAMPHRU2018412; **Taxon:** scientificName: Darevskia valentini; kingdom: Animalia ; phylum: Chordata ; class: Reptilia ; order: Squamata ; family: Lacertidae ; genus: Darevskia ; scientificNameAuthorship: Boettger, 1892; **Location:** country: Armenia; stateProvince: Shirak Province; locality: Mets Sepasar; decimalLatitude: 41.030369; decimalLongitude: 43.820932; geodeticDatum: WGS1984; georeferenceProtocol: GPS; **Identification:** identifiedBy: Arakelyan M.S.; **Event:** samplingProtocol: Captured by noose; eventDate: 2018-7-15; **Record Level:** language: en; rights: https://creativecommons.org/publicdomain/zero/1.0/; rightsHolder: Petrosyan V.G.; accessRights: http://vertnet.org/resources/norms.html; institutionCode: IEERASBIOINF; collectionCode: REPAMPHRU; basisOfRecord: HumanObservation
- **Type status:** Other material. **Occurrence:** catalogNumber: REPAMPHRU2019443; recordedBy: Osipov F.A.; individualCount: 1; sex: fmale; lifeStage: adult; occurrenceID: urn:IEERASBIOINF:REPAMPHRU2019443; **Taxon:** scientificName: Darevskia valentini; kingdom: Animalia ; phylum: Chordata ; class: Reptilia ; order: Squamata ; family: Lacertidae ; genus: Darevskia ; scientificNameAuthorship: Boettger, 1892; **Location:** country: Armenia; stateProvince: Kotayk Province; locality: Tezh; decimalLatitude: 40.6545833333; decimalLongitude: 44.5810166667; geodeticDatum: WGS1984; georeferenceProtocol: GPS; **Identification:** identifiedBy: Arakelyan M.S.; **Event:** samplingProtocol: Captured by noose; eventDate: 2018-7-11; **Record Level:** language: en; rights: https://creativecommons.org/publicdomain/zero/1.0/; rightsHolder: Petrosyan V.G.; accessRights: http://vertnet.org/resources/norms.html; institutionCode: IEERASBIOINF; collectionCode: REPAMPHRU; basisOfRecord: HumanObservation
- **Type status:** Other material. **Occurrence:** catalogNumber: REPAMPHRU2019447; recordedBy: Osipov F.A.; individualCount: 1; sex: fmale; lifeStage: adult; occurrenceID: urn:IEERASBIOINF:REPAMPHRU2019447; **Taxon:** scientificName: Darevskia valentini; kingdom: Animalia ; phylum: Chordata ; class: Reptilia ; order: Squamata ; family: Lacertidae ; genus: Darevskia ; scientificNameAuthorship: Boettger, 1892; **Location:** country: Georgia; stateProvince: Samtskhe-javakheti oblast; locality: Khanchkali lake (Zhdanovkani); decimalLatitude: 41.161321666667; decimalLongitude: 43.794278333333; geodeticDatum: WGS1984; georeferenceProtocol: GPS; **Identification:** identifiedBy: Arakelyan M.S.; **Event:** samplingProtocol: Captured by noose; eventDate: 2019-7-26; **Record Level:** language: en; rights: https://creativecommons.org/publicdomain/zero/1.0/; rightsHolder: Petrosyan V.G.; accessRights: http://vertnet.org/resources/norms.html; institutionCode: IEERASBIOINF; collectionCode: REPAMPHRU; basisOfRecord: HumanObservation
- **Type status:** Other material. **Occurrence:** catalogNumber: REPAMPHRU2019449; recordedBy: Osipov F.A.; individualCount: 1; sex: fmale; lifeStage: adult; occurrenceID: urn:IEERASBIOINF:REPAMPHRU2019449; **Taxon:** scientificName: Darevskia valentini; kingdom: Animalia ; phylum: Chordata ; class: Reptilia ; order: Squamata ; family: Lacertidae ; genus: Darevskia ; scientificNameAuthorship: Boettger, 1892; **Location:** country: Georgia; stateProvince: Samtskhe-Javakheti oblast; locality: Khanchali lake; decimalLatitude: 41.481283333333; decimalLongitude: 43.2802; geodeticDatum: WGS1984; georeferenceProtocol: GPS; **Identification:** identifiedBy: Arakelyan M.S.; **Event:** samplingProtocol: Captured by noose; eventDate: 2019-7-26; **Record Level:** language: en; rights: https://creativecommons.org/publicdomain/zero/1.0/; rightsHolder: Petrosyan V.G.; accessRights: http://vertnet.org/resources/norms.html; institutionCode: IEERASBIOINF; collectionCode: REPAMPHRU; basisOfRecord: HumanObservation
- **Type status:** Other material. **Occurrence:** catalogNumber: REPAMPHRU2019464; recordedBy: Osipov F.A.; individualCount: 1; sex: fmale; lifeStage: adult; occurrenceID: urn:IEERASBIOINF:REPAMPHRU2019464; **Taxon:** scientificName: Darevskia valentini; kingdom: Animalia ; phylum: Chordata ; class: Reptilia ; order: Squamata ; family: Lacertidae ; genus: Darevskia ; scientificNameAuthorship: Boettger, 1892; **Location:** country: Armenia; stateProvince: Kotayk Province; locality: Artavazd; decimalLatitude: 40.620316666667; decimalLongitude: 44.56305; geodeticDatum: WGS1984; georeferenceProtocol: GPS; **Identification:** identifiedBy: Arakelyan M.S.; **Event:** samplingProtocol: Captured by noose; eventDate: 2018-7-14; **Record Level:** language: en; rights: https://creativecommons.org/publicdomain/zero/1.0/; rightsHolder: Petrosyan V.G.; accessRights: http://vertnet.org/resources/norms.html; institutionCode: IEERASBIOINF; collectionCode: REPAMPHRU; basisOfRecord: HumanObservation
- **Type status:** Other material. **Occurrence:** catalogNumber: REPAMPHRU2019465; recordedBy: Osipov F.A.; individualCount: 1; sex: fmale; lifeStage: adult; occurrenceID: urn:IEERASBIOINF:REPAMPHRU2019465; **Taxon:** scientificName: Darevskia valentini; kingdom: Animalia ; phylum: Chordata ; class: Reptilia ; order: Squamata ; family: Lacertidae ; genus: Darevskia ; scientificNameAuthorship: Boettger, 1892; **Location:** country: Georgia; stateProvince: Samtskhe-javakheti oblast; locality: Akhalkalaki (Rio Kirkh-Bulakhi); decimalLatitude: 41.393743333333; decimalLongitude: 43.469711666667; geodeticDatum: WGS1984; georeferenceProtocol: GPS; **Identification:** identifiedBy: Arakelyan M.S.; **Event:** samplingProtocol: Captured by noose; eventDate: 2019-7-26; **Record Level:** language: en; rights: https://creativecommons.org/publicdomain/zero/1.0/; rightsHolder: Petrosyan V.G.; accessRights: http://vertnet.org/resources/norms.html; institutionCode: IEERASBIOINF; collectionCode: REPAMPHRU; basisOfRecord: HumanObservation

#### Notes

**Figure 9.**
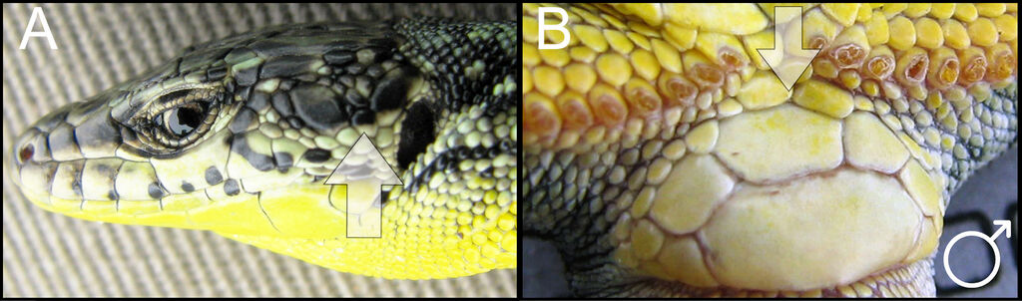
Distinguishing characters of *D. valentini* (male individual on the photographs). **A.** Group of small scales between the central temporal and tympanum shields (indicated by arrow); **B.** Single or sometimes double enlarged pre-anal scale (indicated by arrow). Photos by M. Arakelyan and F. Danielyan.

*Darevskia valentini* is a bisexual “paternal” species for two parthenogenetic *D. armeniaca* and *D. unisexualis* species (Darevsky 1967, Murphy et al. 2000). Species identification in SZs was undertaken by authors at different times using allozyme loci, multilocus DNA fingerprinting, mini- and micro-satellite markers and morphological features (Darevsky 1967, Uzzell and Darevsky 1975, Moritz et al. 1992, Danielyan et al. 2008, Arakelyan et al. 2011, Carretero et al. 2018). In the field, *D. valentini* identification was made using guides according to Darevsky (Darevsky 1967), (Fig. 9). In all nine SZs, 13 individuals were captured, including 11 adult females and two males.

The range of *D. valentini* is divided into several rather vast, but isolated areas, including mountain meadows and mountain steppes of Armenia, Nagorno-Karabakh, southern Georgia and eastern Turkey (Petrosyan et al. 2019a, Petrosyan et al. 2019b). The most extensive areas for its presence cover the mountainous zone of the Geghama Range, which extends to Lake Sevan, in the mountainous region of Aragats, north-western Armenia and the surrounding areas of southern Georgia. During the field survey in 2018-2019, eight SZs were identified with the "daughter" species *D. armeniaca* and two zones with *D. unisexualis*. Hybrid individuals were found in three zones (Lchashen, Kuchak and Tezh), which were previously mentioned in literature (Danielyan et al. 2008, Carretero et al. 2018). The SZs were located on stone bridges along highways, on large stones and clay rocks in the mountain-steppe, mountain meadow subalpine zone of the northern and eastern parts of Armenia, in southern Georgia and on the border with Nagorno-Karabakh.

### Darevskia portschinskii

(Kessler, 1878)

E5C1901D-9391-5279-B790-93CD0A6AB932

#### Materials

- **Type status:** Other material. **Occurrence:** catalogNumber: REPAMPHRU2018396; recordedBy: Osipov F.A.; individualCount: 3; sex: fmale; lifeStage: adult; occurrenceID: urn:IEERASBIOINF:REPAMPHRU2018396; **Taxon:** scientificName: Darevskia portschinskii; kingdom: Animalia ; phylum: Chordata ; class: Reptilia ; order: Squamata ; family: Lacertidae ; genus: Darevskia ; scientificNameAuthorship: Kessler, 1878; **Location:** country: Armenia; stateProvince: Tavush Province; locality: Road to Dilijan - Hagarcin; decimalLatitude: 40.76671; decimalLongitude: 44.919407; geodeticDatum: WGS1984; georeferenceProtocol: GPS; **Identification:** identifiedBy: Arakelyan M.S.; **Event:** samplingProtocol: Captured by noose; eventDate: 2018-7-9; **Record Level:** language: en; rights: https://creativecommons.org/publicdomain/zero/1.0/; rightsHolder: Petrosyan V.G.; accessRights: http://vertnet.org/resources/norms.html; institutionCode: IEERASBIOINF; collectionCode: REPAMPHRU; basisOfRecord: HumanObservation
- **Type status:** Other material. **Occurrence:** catalogNumber: REPAMPHRU2019427; recordedBy: Osipov F.A.; individualCount: 1; sex: male; lifeStage: adult; occurrenceID: urn:IEERASBIOINF:REPAMPHRU2019427; **Taxon:** scientificName: Darevskia portschinskii; kingdom: Animalia ; phylum: Chordata ; class: Reptilia ; order: Squamata ; family: Lacertidae ; genus: Darevskia ; scientificNameAuthorship: Kessler, 1878; **Location:** country: Armenia; stateProvince: Lori Province; locality: Dzoraget; decimalLatitude: 41.014219; decimalLongitude: 44.379631; geodeticDatum: WGS1984; georeferenceProtocol: GPS; **Identification:** identifiedBy: Arakelyan M.S.; **Event:** samplingProtocol: Captured by noose; eventDate: 2019-7-19; **Record Level:** language: en; rights: https://creativecommons.org/publicdomain/zero/1.0/; rightsHolder: Petrosyan V.G.; accessRights: http://vertnet.org/resources/norms.html; institutionCode: IEERASBIOINF; collectionCode: REPAMPHRU; basisOfRecord: HumanObservation
- **Type status:** Other material. **Occurrence:** catalogNumber: REPAMPHRU2019445; recordedBy: Osipov F.A.; individualCount: 2; sex: fmale; lifeStage: adult; occurrenceID: urn:IEERASBIOINF:REPAMPHRU2019445; **Taxon:** scientificName: Darevskia portschinskii; kingdom: Animalia ; phylum: Chordata ; class: Reptilia ; order: Squamata ; family: Lacertidae ; genus: Darevskia ; scientificNameAuthorship: Kessler, 1878; **Location:** country: Armenia; stateProvince: Tavush Province; locality: Dilidjan forest; decimalLatitude: 40.757211666667; decimalLongitude: 44.804148333333; geodeticDatum: WGS1984; georeferenceProtocol: GPS; **Identification:** identifiedBy: Arakelyan M.S.; **Event:** samplingProtocol: Captured by noose; eventDate: 2019-7-22; **Record Level:** language: en; rights: https://creativecommons.org/publicdomain/zero/1.0/; rightsHolder: Petrosyan V.G.; accessRights: http://vertnet.org/resources/norms.html; institutionCode: IEERASBIOINF; collectionCode: REPAMPHRU; basisOfRecord: HumanObservation
- **Type status:** Other material. **Occurrence:** catalogNumber: REPAMPHRU2019451; recordedBy: Osipov F.A.; individualCount: 4; sex: fmale; lifeStage: adult; occurrenceID: urn:IEERASBIOINF:REPAMPHRU2019451; **Taxon:** scientificName: Darevskia portschinskii; kingdom: Animalia ; phylum: Chordata ; class: Reptilia ; order: Squamata ; family: Lacertidae ; genus: Darevskia ; scientificNameAuthorship: Kessler, 1878; **Location:** country: Nagorno-Karabakh; stateProvince: Shahumyan Province; locality: Zuar; decimalLatitude: 40.047933333333; decimalLongitude: 46.2367; geodeticDatum: WGS1984; georeferenceProtocol: GPS; **Identification:** identifiedBy: Arakelyan M.S.; **Event:** samplingProtocol: Captured by noose; eventDate: 2018-7-10; **Record Level:** language: en; rights: https://creativecommons.org/publicdomain/zero/1.0/; rightsHolder: Petrosyan V.G.; accessRights: http://vertnet.org/resources/norms.html; institutionCode: IEERASBIOINF; collectionCode: REPAMPHRU; basisOfRecord: HumanObservation
- **Type status:** Other material. **Occurrence:** catalogNumber: REPAMPHRU2019453; recordedBy: Osipov F.A.; individualCount: 2; sex: fmale; lifeStage: adult; occurrenceID: urn:IEERASBIOINF:REPAMPHRU2019453; **Taxon:** scientificName: Darevskia portschinskii; kingdom: Animalia ; phylum: Chordata ; class: Reptilia ; order: Squamata ; family: Lacertidae ; genus: Darevskia ; scientificNameAuthorship: Kessler, 1878; **Location:** country: Armenia; stateProvince: Tavush Province; locality: Gosh; decimalLatitude: 40.73916667; decimalLongitude: 45.02076667; geodeticDatum: WGS1984; georeferenceProtocol: GPS; **Identification:** identifiedBy: Arakelyan M.S.; **Event:** samplingProtocol: Captured by noose; eventDate: 2018-7-21; **Record Level:** language: en; rights: https://creativecommons.org/publicdomain/zero/1.0/; rightsHolder: Petrosyan V.G.; accessRights: http://vertnet.org/resources/norms.html; institutionCode: IEERASBIOINF; collectionCode: REPAMPHRU; basisOfRecord: HumanObservation
- **Type status:** Other material. **Occurrence:** catalogNumber: REPAMPHRU2019461; recordedBy: Osipov F.A.; individualCount: 1; sex: male; lifeStage: adult; occurrenceID: urn:IEERASBIOINF:REPAMPHRU2019461; **Taxon:** scientificName: Darevskia portschinskii; kingdom: Animalia ; phylum: Chordata ; class: Reptilia ; order: Squamata ; family: Lacertidae ; genus: Darevskia ; scientificNameAuthorship: Kessler, 1878; **Location:** country: Nagorno-Karabakh; stateProvince: Shahumyan Province; locality: Road to Karvachar; decimalLatitude: 40.096783333333; decimalLongitude: 46.0338; geodeticDatum: WGS1984; georeferenceProtocol: GPS; **Identification:** identifiedBy: Arakelyan M.S.; **Event:** samplingProtocol: Captured by noose; eventDate: 2018-7-10; **Record Level:** language: en; rights: https://creativecommons.org/publicdomain/zero/1.0/; rightsHolder: Petrosyan V.G.; accessRights: http://vertnet.org/resources/norms.html; institutionCode: IEERASBIOINF; collectionCode: REPAMPHRU; basisOfRecord: HumanObservation
- **Type status:** Other material. **Occurrence:** catalogNumber: REPAMPHRU2019467; recordedBy: Osipov F.A.; individualCount: 1; sex: fmale; lifeStage: adult; occurrenceID: urn:IEERASBIOINF:REPAMPHRU2019467; **Taxon:** scientificName: Darevskia portschinskii; kingdom: Animalia ; phylum: Chordata ; class: Reptilia ; order: Squamata ; family: Lacertidae ; genus: Darevskia ; scientificNameAuthorship: Kessler, 1878; **Location:** country: Georgia; stateProvince: Kvemo Kartli region; locality: Guguti; decimalLatitude: 41.200509; decimalLongitude: 44.552717; geodeticDatum: WGS1984; georeferenceProtocol: GPS; **Identification:** identifiedBy: Arakelyan M.S.; **Event:** samplingProtocol: Captured by noose; eventDate: 2019-6-18; **Record Level:** language: en; rights: https://creativecommons.org/publicdomain/zero/1.0/; rightsHolder: Petrosyan V.G.; accessRights: http://vertnet.org/resources/norms.html; institutionCode: IEERASBIOINF; collectionCode: REPAMPHRU; basisOfRecord: HumanObservation
- **Type status:** Other material. **Occurrence:** catalogNumber: REPAMPHRU2019469; recordedBy: Osipov F.A.; individualCount: 1; sex: fmale; lifeStage: adult; occurrenceID: urn:IEERASBIOINF:REPAMPHRU2019469; **Taxon:** scientificName: Darevskia portschinskii; kingdom: Animalia ; phylum: Chordata ; class: Reptilia ; order: Squamata ; family: Lacertidae ; genus: Darevskia ; scientificNameAuthorship: Kessler, 1878; **Location:** country: Armenia; stateProvince: Lori Province; locality: Karmir Ageg; decimalLatitude: 40.97993; decimalLongitude: 44.56121; geodeticDatum: WGS1984; georeferenceProtocol: GPS; **Identification:** identifiedBy: Arakelyan M.S.; **Event:** samplingProtocol: Captured by noose; eventDate: 2019-6-18; **Record Level:** language: en; rights: https://creativecommons.org/publicdomain/zero/1.0/; rightsHolder: Petrosyan V.G.; accessRights: http://vertnet.org/resources/norms.html; institutionCode: IEERASBIOINF; collectionCode: REPAMPHRU; basisOfRecord: HumanObservation
- **Type status:** Other material. **Occurrence:** catalogNumber: REPAMPHRU2019471; recordedBy: Osipov F.A.; individualCount: 1; sex: fmale; lifeStage: adult; occurrenceID: urn:IEERASBIOINF:REPAMPHRU2019471; **Taxon:** scientificName: Darevskia portschinskii; kingdom: Animalia ; phylum: Chordata ; class: Reptilia ; order: Squamata ; family: Lacertidae ; genus: Darevskia ; scientificNameAuthorship: Kessler, 1878; **Location:** country: Armenia; stateProvince: Lori Province; locality: Privolnoe; decimalLatitude: 41.148065; decimalLongitude: 44.466437; geodeticDatum: WGS1984; georeferenceProtocol: GPS; **Identification:** identifiedBy: Arakelyan M.S.; **Event:** samplingProtocol: Captured by noose; eventDate: 2019-6-18; **Record Level:** language: en; rights: https://creativecommons.org/publicdomain/zero/1.0/; rightsHolder: Petrosyan V.G.; accessRights: http://vertnet.org/resources/norms.html; institutionCode: IEERASBIOINF; collectionCode: REPAMPHRU; basisOfRecord: HumanObservation

#### Notes

**Figure 10.**
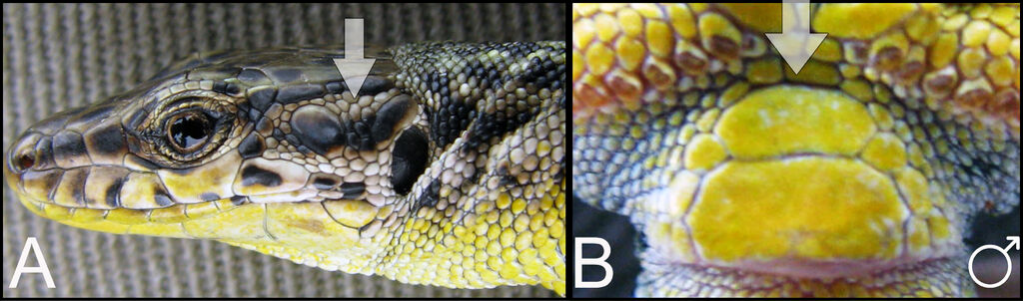
Distinguishing characters of *D. portschinskii* (male individual on the photographs). **A.** Central temporal shield is medium size, small or not explicit, from the first upper temporal shield, it is divided by 1-5 longitudinal rows of small shields (indicated by arrow); **B.** Anal shield is large, elongated across; in front of it, one large, more or less rounded at the posterior margin pre-anal shield is located symmetrically (indicated by arrow). Photos by M. Arakelyan and F. Danielyan.

*Darevskia portschinskii* is a bisexual “paternal” species for two parthenogenetic forms, *D. dahli* and *D. rostombekowi* (Darevsky 1967, Uzzell and Darevsky 1975, Murphy et al. 2000). Species identification in the SZs was carried out by different authors using allozyme markers, multilocus DNA fingerprinting, mini- and micro-satellite markers and morphological features (Moritz et al. 1992, MacCulloch et al. 1997, Tokarskaya et al. 2001, Arakelyan et al. 2011, Vergun et al. 2014). In the field surveys, species identification was carried out using identification guides (Darevsky 1967) (Fig. 10). In all nine zones, 16 individuals were captured, including 14 adult females and two males.

The range of *D. portschinskii* covers the valleys of the middle reaches of the Kura River within central and southern Georgia, northern Armenia and north-western Azerbaijan (Darevsky 1967, Tarkhnishvili et al. 2010,Petrosyan et al. 2020). The range in Armenia begins in the vicinity of Stepanavan in the west and is limited in the east by the Sevan Ridge. Suitable habitats in Azerbaijan cover the border territories with Armenia to the valley of the middle reaches of the river Ganjachaya. The presence of the species in the territory of Nagorno-Karabakh was previously mentioned in literature (Arakelyan et al. 2011). Within the study area, we identified six SZs in the two Provinces of Armenia (Tavush, Lori), two zones in Nagorno-Karabakh and one in Georgia. *Darevskia portschinskii* co-exist in three SZs with “daughter” species *D. dahli* and *D. rostombekowi* in two SZs. Two parenteal bisexual species *D. portschinskii* and *D. r. raddei* co-exist in two SZs (Uzzell and Darevsky 1975, Galoyan et al. 2019, Galoyan et al. 2020). All zones are mainly located in the lower band of mountain forests in relatively warm and arid forest and shrub biotopes.

## Analysis

**Figure 11.**
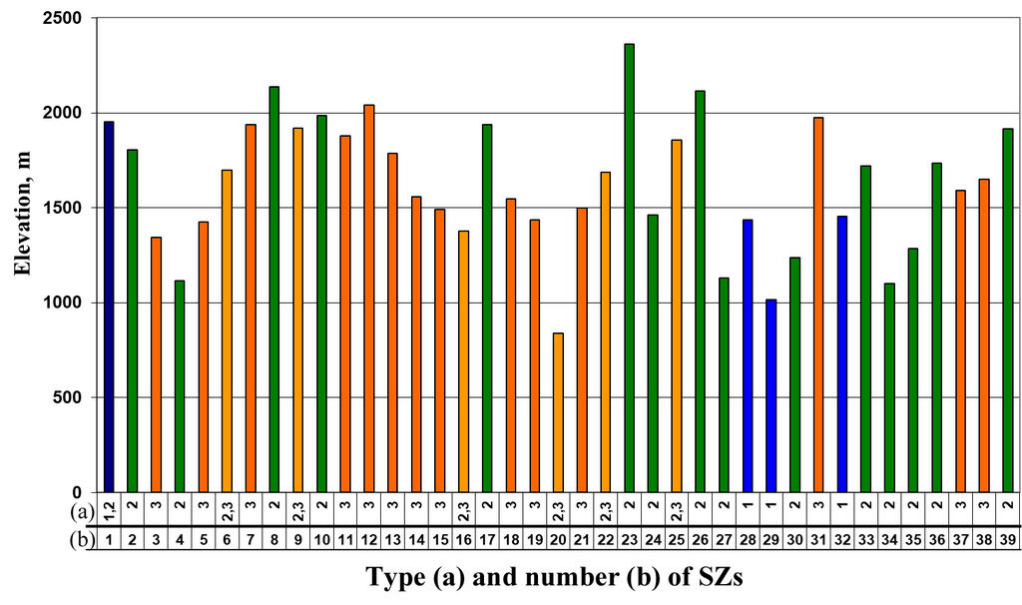
Altitudinal distribution of SZs of parental and parthenogenetic species of lizards of the genus *Darevskia*. Type of SZs (a): 1- between parental bisexual species, 2 – between parental and parthenogenetic species; 3 – between parthenogenetic species. The geographic location of SZs (b) is shown in Fig. 1.

An analysis of the altitudinal position of SZs shows that they are located in a range from 837 m to 2360 m above sea level (mean ± SE = 1623 ± 55 m), (Fig. 11). Altitudinal distribution of SZs of bisexual (*D. r. nairensis - D. valentini; D. portschinskii - D. r. raddei*), bisexual - parthenogenetic, and parthenogenetic species are characterised by the following positions above sea level – 1952 m (n = 1), 1302 ± 199 m (n = 3); 1561 ± 141 m (n = 6); 1654 ± 92 m (n = 14), respectively. Additional analysis separately for parthenogenetic species shows that *D. unisexualis* is present in SZs at an altitude of 1847 ± 96 m (n = 11), *D. armeniaca* - 1736 ± 61 m (n = 24); *D. dahli* - 1504 ± 78 m (n = 15) and *D. rostombekowi* - 1384 ± 91 m (n = 10). The analysis of the altitudinal distribution of SZs for the studied species shows that, in general, the statement is true that the possibilities for hybridisation appear to be due to the penetration of male bisexual species into the range of parthenogenetic species. At high altitudes of 1564–2252 m (mean ± SE = 1908 ± 115 m, n = 8), this penetration is associated with males of the paternal species *D. valentini* for *D. armeniaca* and *D. unisexualis* and, at low altitudes of 1106–1556 m above sea level (mean ± SE = 1331 ± 75, n = 9), it is associated with males of another paternal species, *D. portschinskii* for *D. dahli* and *D. rostombekowi*, respectively. These new data refine and enlarge the estimates presented in literature (Danielyan et al. 2008), which records that hybridisation zones in Armenia exist in the mountainous regions of Central Armenia at altitudes from 1800 m to 2000 m above sea level.

## Discussion

We identified new SZs and refined and determined the geographical and altitudinal distribution parameters of previously-known zones during the field survey in 2018-2019. Although most of the 39 SZs were previously known in literature, nevertheless, during the field surveys, we confirmed that they really were SZs at the present time. We found five previously-unknown SZs: № 12 (Tsilkar, Aragatsotn Province, 40.736893°N, 44.197427°E), № 19 (close to city Ijevan, 40.868307°N, 45.187475°E), № 22 (Keti, Shirak Province, 40.864013°N, 43.841847°E), №35 (Karmir Ageg, Lori Province, 40.97993°N, 44.56121°E) and № 37 (Dorbantvank, Lori Province, 41.113712°N, 44.435583°E). From Table 1, the number of SZs of bisexual parental species is small and equal to four: № 1 (Lchashen, Gegharkunik Province, 40.510698°N, 44.935422°E); № 28 (Zuar, Nagorno-Karabakh, Shahumyan Province, 40.04793333°N, 46.2367°E); № 29 (Gosh, Tavush Province, 40.73916667°N, 45.02076667°E); № 32 (Kelbadzhar, Nagorno-Karabakh, Shahumyan Province, 40.09678333°N, 46.0338°E). The processes taking place in these zones between bisexual parental rock lizard species are of particular interest, since interspecific hybridisation of these species has given rise to parthenogenetic species (Darevsky 1966, Borkin and Darevsky 1980, Arakelyan and Danielyan 2014).

Generalisation of our new and published data showed that, at present, SZs of *D. valentini* and *D. mixta*, as well as *D. portschinskii* and *D. mixta*, could not be found which, in the past, gave rise to parthenogenetic species *D. armeniaca* and *D. dahli*, respectively (Tarkhnishvili et al. 2010, Petrosyan et al. 2019a). Recently, Tarkhnishvili et al. (2017) suggested new hypothesis of origin of *D. armeniaca*. According to this hypothesis, it arose from backcrosses of male *D. valentini* with parthenogenetic *D. dahli* (Tarkhnishvili et al. 2017). However, additional studies are needed to determine which of two or both scenarios of *D. armeniaca* origin have matter.

A pair of parental species *D. valentini* and *D. r. raddei* may form a SZ in western Turkey, but we do not have data on the processes taking place there due to poor knowledge of this region. In the SZ of *D. valentini* and *D. r. nairensis* (№ 1, Lchashen, Sevan Lake) in the vicinity of the village of Lchashen, where highland "paternal" species *D. valentini* penetrate into the range of *D. r. nairensis*, intermediate forms are not formed and individuals of both species have no combined characteristics. This is due to reproductive isolation as a result of the maturation of gonads at different times (Danielyan 1965, Arakelyan 2012).

In addition, there are three other SZs of *D. r. raddei* and *D. portschinskii* (№ 28, 29, 32). The widest overlap is observed in SZ № 28 (Zuar, Shahumyan Province), located in the valley of the river Tutun in the Shahumyan Province of Nagorno-Karabakh. Here, landscapes are represented by rock outcrops along the road in the mountain deciduous forest zone. In more arid biotopes, these are inhabited only by *D. r. raddei* and, in more darkened areas of the forest, *D. portschinskii* is more common. The composition of the mixed population consists of 41.5% *D. portschinskii*, 43.1% - *D. r. raddei* and 15.4% - hybrid individuals, which, according to morphological characteristics, cannot be attributed to either of two species (Arakelyan and Danielyan 2014), although the latest behavioural, morphological and microsatellite studies did not support the presence of the hybrid individuals (Galoyan et al. 2020). Our analysis suggests that there is an important SZ № 28 (Zuar, Shahumyan Province) at the present time, where intensive hybridisation processes may occur between bisexual species. These species belong to the clades “rudis” and “caucasica” and they are capable of giving rise to parthenogenetic species (Arakelyan 2012).

In SZ № 29, Gosh, Tavush Province, since the 1960s study of the populations, single hybrid individuals with mixed signs of folidosis and colour have been found (Danielyan 1989). The absence of hybrid individuals was additionally confirmed in 1973 (Uzzel and Darevsky 1973). However, a further change in the composition of the mixed population was found in the period 2005-2011, where the number of *D. portschinskii* was significantly reduced and no hybrid individuals were found. Amongst 64 captured lizards, only six (9%) individuals were *D. portschinskii* and the rest were *D. r. raddei* (Arakelyan 2012). In SZ № 29 (Gosh, Tavush Province), the separation of ecological niches has occurred over time and, despite the possibility of hybridisation, the proportion of hybrids sharply decreased here due to the absence of contact zones between the two species.

In the SZs of the second type (№ 1, 2, 4, 6, 8-10, 16, 17, 20, 22-27, 30, 33-36, 39) (Table 1), triploid hybrids can potentially be expected to result from spontaneous hybridisation between males of bisexual species and parthenogenetic forms (Fig. 1, Table 1). The findings of triploid hybrids were widely mentioned in a number of previous publications (Darevsky and Kulikova 1962, Darevsky and Kulikova 1964, Borkin and Darevsky 1980, Danielyan et al. 2008, Arakelyan 2012, Carretero et al. 2018). In addition, there are possibilities of mating of rare males produced by a parthenogenetic lineage with females of bisexual species, i.e. the possibility of developing contagious parthenogenesis (Maccari et al. 2014).

The zones of the third type (№ 3, 5-7, 9, 11-16, 18-22, 25, 31, 37, 38) include mixed populations of several parthenogenetic species. The formation of new hybrids as a result of crossbreeding of rare parthenogenetic males produced by a parthenogenetic lineage with females of another parthenogenetic species was presented (Danielyan 1987). This paper presents the results of the analysis of SZ № 3 (Dilijan, Tavush Province, 40.733998° N, 44.81778° E) of three parthenogenetic species *D. armeniaca*, *D. dahli* and *D. rostombekowi*. This SZ is of particular interest, since, amongst the *D. dahli*, individual lizards with a bright yellow colour on the lower side of the body were found, which the authors conventionally called “yellow” *D. dahli*. These individuals differed sharply in colour from those of the usual *D. dahli*. For comparative analysis of folidosis (15 features), 63 lizards were used, of which 19 belonged to the usual *D. dahli*, 23 to the “yellow” *D. dahli* and 21 to *D. rostombekowi*. A comparative analysis of the characters in these parthenogenetic lizards showed that, according to seven features, the “yellow” *D. dahli* occupies an intermediate position between the common *D. dahli* and *D. rostombekowi.* For example, by the number of scales around the middle of the body in one row, the “yellow” *D. dahli* coincides with *D. rostombekowi* by 37.5%, with the common *D. dahli* by 50%, but differs from both species by 12.5%. Experimental data showed that the intermediate position of the "yellow" *D. dahli* is also confirmed by fecundity. If the clutch of common *D. dahli* consisted of 2-5 eggs and the clutch of *D. rostombekowi* of two, less than three, then the “yellow” *D. dahli* had at least three eggs in the clutch. These forms were also studied by skin transplantation methods to analyse tissue compatibility between *D. rostombekowi*, common and "yellow" *D. dahli*. A comprehensive analysis made it possible to confirm that a male *D. dahli* or *D. rostombekowi* was produced in the studied populations, which then crossbred with females of *D. rostombekowi* or *D. dahli*. As a result of this crossbreeding, "yellow" individuals of *D. dahli* were formed, which later began to breed parthenogenetically, producing similar "yellow" individuals of *D. dahli*. These results suggest that the formation of new hybrid forms is not excluded in these zones, i.e. mating of rare males produced by a parthenogenetic lineage with females other parthenogenetic forms.

New findings expand our knowlege of geographical distribution of the SZs of unisexual and bisexal parental species, providing a basis for studying reticulate evolution and hybridogeneous speciation (Borkin and Darevsky 1980, Moritz 1991). Our comprehensive analysis of museum collection specimens, monographs and articles showed that false or ambiguous records of the distribution of SZ mainly arose due to various reasons. Basically, these were inaccurate due to changes in the name of taxa, fuzzy descriptions of places for collecting lizards in settlements, lack of coordinates of species occurrence points and names of geographical projections of maps, which led to incorrect interpretation of geographical data. A thorough study of SZs, based on the collection of the data on the exact finding of species, is the only way to obtain reliable information on the SZs and to understand the mechanisms of reticulate evolution in the past, present and future.

## Supplementary Material

XML Treatment for Darevskia armeniaca

XML Treatment for Darevskia dahli

XML Treatment for Darevskia rostombekowi

XML Treatment for Darevskia unisexualis

XML Treatment for Darevskia raddei raddei

XML Treatment for Darevskia raddei nairensis

XML Treatment for Darevskia valentini

XML Treatment for Darevskia portschinskii

## Figures and Tables

**Table 1.** List of SZs in the Armenia and adjacent territories, where the type of SZ are: 1 is SZ of parental bisexual species, 2 is SZ of parental species with unisexual species, 3 is SZ of the parthenogenetic species.

**SZ number**	**Type of SZ**	**Partenogenetic species**	**Bisexual species**	**Decilmial longitude, Decimal latitude**
1	1,2	*D. armeniaca*	*D. r. nairensis*, *D. valentini*	44.9354, 40.5107
2	2	*D. unisexualis*	*D. r. nairensis*	44.6020, 40.6092
3	3	*D. armeniaca*, *D. dahli*, *D. rostombekowi*		44.8178, 40.7340
4	2	*D. rostombekowi*	*D. portschinskii*	44.9194, 40.7667
5	3	*D. armeniaca*, *D. rostombekowi*		44.8906, 40.8019
6	2,3	*D. armeniaca*, *D. r. nairensis*, *D. unisexualis*	*D. r. nairensis*	44.7488, 40.5064
7	3	*D. rostombekowi*, *D. unisexualis*		45.6240, 40.1857
8	2	*D. armeniaca*	*D. valentini*	46.0010, 40.2231
9	2,3	*D. armeniaca*, *D. unisexualis*	*D. valentini*	44.3884, 40.5287
10	2	*D. armeniaca*	*D. valentini*	43.8209, 41.0304
11	3	*D. armeniaca*, *D. dahli*		44.2024, 40.8954
12	3	*D. armeniaca*, *D. dahli*, *D. unisexualis*		44.1974, 40.7369
13	3	*D. armeniaca*, *D. dahli*		44.4367, 40.9173
14	3	*D. armeniaca*, *D. dahli*		44.4402, 40.9328
15	3	*D. armeniaca*, *D. dahli*		44.4793, 40.9385
16	2,3	*D. armeniaca*, *D. dahli*	*D. portschinskii*	44.3796, 41.0142
17	2	*D. unisexualis*	*D. r. nairensis*	45.1444, 40.3972
18	3	*D. rostombekowi*, *D. unisexualis*		44.2775, 40.8249
19	3	*D. armeniaca*, *D. dahli*, *D. rostombekowi*		45.1875, 40.8683
20	2,3	*D. dahli*, *D. rostombekowi*	*D. r. raddei*	45.0526, 41.0263
21	3	*D. armeniaca*, *D. dahli*, *D. rostombekowi*		44.7706, 40.7156
22	2,3	*D. dahli*, *D. unisexualis*	*D. r. nairensis*	43.8418, 40.8640
23	2	*D. armeniaca*	*D. valentini*	44.5810, 40.6546
24	2	*D. rostombekowi*	*D. portschinskii*	44.8041, 40.7572
25	2,3	*D. armeniaca*, *D. unisexualis*	*D. valentini*	44.5631, 40.6203
26	2	*D. armeniaca*	*D. valentini*	43.7943, 41.1613
27	2	*D. armeniaca*	*D. valentini*	43.2802, 41.4813
28	1		*D. portschinskii*, *D. r. raddei*	46.2367, 40.0479
29	1		*D. portschinskii*, *D. r. raddei*	45.0208, 40.7392
30	2	*D. rostombekowi*	*D. r. raddei*	44.9971, 40.7299
31	3	*D. armeniaca*, *D. unisexualis*		44.6972, 40.5352
32	1		*D. r. raddei*, *D. portschinskii*	46.0338, 40.0968
33	2	*D. armeniaca*	*D. valentini*	43.4697, 41.3937
34	2	*D. dahli*	*D. portschinskii*	44.5527, 41.2005
35	2	*D. dahli*	*D. portschinskii*	44.5612, 40.9799
36	2	*D. armeniaca*	*D. portschinskii*	44.4664, 41.1481
37	3	*D. armeniaca*, *D. dahli*		44.4356, 41.1137
38	3	*D. armeniaca*, *D. dahli*		44.3089, 41.1546
39	2	*D. unisexualis*	*D. r. nairensis*	45.0621, 40.4673
